# Association of post-stroke fatigue with physical activity and physical fitness: A systematic review and meta-analysis

**DOI:** 10.1177/17474930231152132

**Published:** 2023-02-03

**Authors:** Petra Larsson, Julia Bidonde, Unni Olsen, Caryl L Gay, Anners Lerdal, Marie Ursin, Gillian Elizabeth Mead, Elisabeth Edvardsen

**Affiliations:** 1 Department of Interdisciplinary Health Sciences, Institute of Health and Society, Faculty of Medicine, University of Oslo, Oslo, Norway; 2 Surgical Department, Lovisenberg Diaconal Hospital, Oslo, Norway; 3 Division for Health Services, Norwegian Institute of Public Health, Oslo, Norway; 4 School of Rehabilitation Science, University of Saskatchewan, Saskatoon, Canada; 5 Department of Public Health Science, Institute of Health and Society, Faculty of Medicine, University of Oslo, Oslo, Norway; 6 Department of Family Health Care Nursing, University of California, San Francisco, CA, USA; 7 Research Department, Lovisenberg Diaconal Hospital, Oslo, Norway; 8 Department of Medical Research, Bærum Hospital, Vestre Viken Hospital Trust, Gjettum, Norway; 9 Geriatric Medicine, Division of Health Sciences, Centre for Clinical Brain Sciences, The University of Edinburgh, Edinburgh, UK; 10 Department of Physical Performance, Norwegian School of Sport Sciences, Oslo, Norway; 11 Department of Pulmonary Medicine, Oslo University Hospital, Oslo, Norway

**Keywords:** Associations, meta-analysis, physical activity, physical fitness, post-stroke fatigue, systematic review

## Abstract

**Background:**

It has been hypothesized that post-stroke fatigue (PSF) is associated with reduced physical activity (PA) and impaired physical fitness (fitness). Understanding associations between PSF and PA, and/or fitness could help guide the development of targeted exercise interventions to treat PSF.

**Aims:**

Our systematic review and meta-analysis aimed to investigate PSF’s associations with PA and fitness.

**Summary of review:**

Following a registered protocol, we included studies with cross-sectional or prospective observational designs, published in English or a Scandinavian language, which reported an association of PSF with PA and/or fitness in adult stroke survivors. We searched MEDLINE, Embase, AMED, CINAHL, PsycINFO, ClinicalTrials.gov, and World Health Organization’s International Clinical Trials Registry Platform from inception to November 30, 2022. Risk of bias was assessed using Quality in Prognosis Studies. Thirty-two unique studies (total n = 4721 participants, 55% male), and three study protocols were included. We used random-effects meta-analysis to pool data for PA and fitness outcomes, and vote-counting of direction of association to synthesize data that could not be meta-analyzed. We found moderate-certainty evidence of a weak association between higher PSF and impaired fitness (meta *r* = –0.24; 95% confidence interval (CI) = –0.33, –0.15; n = 905, 7 studies), and very low-certainty evidence of no association between PSF and PA (meta *r* = –0.09; 95% CI = –0.34, 0.161; n = 430, 3 studies). Vote-counting showed a higher proportion of studies with associations between higher PSF and impaired fitness (pˆ = 0.83; 95% CI = 0.44, 0.97; p = 0.22, n = 298, 6 studies), and with associations between higher PSF and lower PA (pˆ = 0.75; 95% CI = 0.51, 0.90; p = 0.08, n = 2566, 16 studies). Very low- to moderate-certainty evidence reflects small study sample sizes, high risk of bias, and inconsistent results.

**Conclusions:**

The meta-analysis showed moderate-certainty evidence of an association between higher PSF and impaired fitness. These results indicate that fitness might protect against PSF. Larger prospective studies and randomized controlled trials evaluating the effect of exercise on PSF are needed to confirm these findings.

## Introduction

Post-stroke fatigue (PSF) is a debilitating condition that affects rehabilitation outcomes,^1^ quality of life,^2^ and mortality.^1^ The reported prevalence of PSF ranges from 25 to 85%.^3^ PSF’s etiology is unknown, but is believed to be multifactorial and different types may exist.^4^

The literature suggests that PSF could be associated with physical activity (PA) and physical fitness (fitness),^5^ which are two distinct, yet related outcomes. PA is defined as “any bodily movement produced by skeletal muscles that results in energy expenditure,”^6^ while fitness is “a set of attributes that people have or achieve that relates to the ability to perform PA.”^6^ Components of fitness include, but are not restricted to, aerobic fitness, muscle endurance, muscle strength, and body composition.^6^ PSF may lead to reduced PA and consequently to impaired fitness, but it is also possible that impaired fitness following a stroke may trigger PSF, leading to reduced PA and further impairments in fitness.^5^ PA and fitness are both modifiable factors, so that, although causal relationships are difficult to establish, a better understanding of their associations with PSF could guide development of targeted exercise interventions to reduce PSF.^5^ This would be an important step, as there are currently no established interventions for treating PSF, although exercise training interventions have been successful in decreasing fatigue in other conditions.^7,8^ A systematic review on associations between PSF and impaired fitness concluded that the three eligible studies yielded insufficient evidence about any such association.^5^ As additional studies have since been published, we have updated and expanded this review using broader inclusion criteria.

Our primary aim was to conduct a systematic review and meta-analysis to determine whether PSF is associated with PA and/or fitness in patients with stroke. Our secondary aim was to explore PSF’s associations across different sub-domains of PA and fitness.

## Methods

The study protocol was registered in the Prospective Register of Systematic Reviews (PROSPERO; CRD42021216435) and reported in accordance with the Preferred Reporting Items for Systematic Review and Meta-analysis (PRISMA) statement.^9^

### Eligibility criteria

Eligible studies had cross-sectional or prospective observational designs, were published in English or a Scandinavian language, included stroke survivors aged 18 years or older, and examined associations between PSF and PA and/or fitness. Studies that only measured balance, activities of daily living, or functional limitations were excluded. We included published study protocols/clinical trial registry records (TRRs)) if they met our inclusion criteria.

### Search strategies and selection criteria

We performed a systematic search in AMED, CINAHL, Embase, MEDLINE, PsychINFO, ClinicalTrials.gov, and World Health Organization’s (WHO) International Clinical Trials Registry Platform (ICTRP) from inception to November 30, 2022. The search strategies are described in Figure S1.

We de-duplicated records in EndNote X9^10^ and imported them into DistillerSR^11^ or Rayyan.^12^ Two authors (PL and EE) independently screened titles and abstracts, and full-text articles using a pre-piloted eligibility-criteria checklist. TRRs were screened in Rayyan or manually by one author (PL).

### Data extraction

Two authors (PL and EE) independently extracted data from eligible studies using a customized data extraction form in DistillerSR. For the updated search, PL extracted the data and another author (MU or AL) cross-checked the extraction. If disagreements could not be resolved, a third author (JB) was consulted. Data included publication details, study and patient characteristics, PSF outcome, PA/fitness outcome, and measure of association (Table S1). We contacted study authors if additional information was needed. Data from multiple reports of the same study were linked together and treated as one. For each study, only one association per outcome (i.e. PA/fitness) was extracted. If a study reported more than one association per outcome, we chose the most relevant (Table S2).

### Data synthesis

We performed a meta-analysis when two or more studies reported a correlational value between PSF and PA, or PSF and fitness. We used Comprehensive Meta-analysis software^13^ to calculate meta correlation (meta *r*), 95% confidence intervals (CIs), and prediction intervals. We used random-effects models, which assume that study population or design differences may influence the data.^14^ Heterogeneity was assessed by prediction intervals^15^ and by visually inspecting the Forest plot. We could not perform sub-group and sensitivity analyses predefined in the PROSPERO protocol, due to an insufficient number of studies. Data that could not be pooled were synthesized by vote-counting of direction of association. We calculated the proportion of studies reporting a negative association (higher PSF and lower PA/fitness) along with 95% CIs (Wilson intervals) using an online calculator.^16^ A two-sided p-value was obtained from the binomial probability test using Stata software.^17^ Vote-counting does not consider statistical significance or magnitude of association.^14^

### Risk of bias assessment

Two authors (EE and PL) independently assessed risk of bias. If consensus was not reached, a third author (JB) was consulted. We used the Quality In Prognosis Studies (QUIPS) checklist,^18^ modified for our study. QUIPS has six domains (Table S3), each rated as having low, moderate, or high risk of bias.

To detect outcome-reporting biases, we searched for existing protocols for the included studies. We compared the planned outcomes reported in the protocols with reported outcomes in the final publications.

### Certainty of evidence

One author (PL) rated certainty of evidence and a second author (JB) cross-checked the rating using the Grading of Recommendations, Assessment, Development and Evaluations (GRADE) framework.^19^ Quality of evidence was rated as very low, low, moderate, or high. We summarized the evidence in GRADEpro.^20^

## Results

### Search results

The search identified 4125 records. One additional record was obtained from the reference list of a systematic review.^5^ After duplicates were removed, 2298 records remained for screening. We excluded 2146 records based on titles and abstracts. We assessed 285 TRRs for eligibility in Rayyan, 40 TRRs manually, and 153 full-text documents in DistillerSR/Rayyan. Thirty-four published papers and two TRRs met the inclusion criteria (Figure S2).

### Study characteristics

We included 32 unique studies (total n = 4721 participants, 55% males). Michael and colleagues published two articles^21,22^ on the same sample, both referred to as Michael 2007.^21^ One published protocol and one TRR described included studies.^23,24^ The second TRR was ongoing.^25^ Most studies were of cross-sectional design (n = 22), the others were longitudinal prospective studies (n = 7) or randomized controlled trials (RCTs; n = 2) (Table S4). All studies were published in English between 2006 and 2022. Table S5 describes the characteristics of each included study.

### Outcomes

We extracted data for 34 PSF associations, 20 with PA^21,23,26–43^ and 14 with fitness.^21,24,31,32,44–53^ Two studies^21,31^ contributed an association for both outcomes and the rest for only one (n = 30). Three studies became eligible once the authors provided additional information regarding the regression coefficient^39^ or direction of association.^40,41^ The nine-item Fatigue Severity Scale (FSS-9) (19 studies), step-count (six studies), and gait-speed (six studies) were the most frequently used outcome measures for PSF, PA, and fitness, respectively (Table S4). Meta-analyses were performed for PSF and PA (three studies)^21,38,41^ and for PSF and fitness (seven studies).^46–49,51,52,54^ There were not enough similar studies to perform meta-analyses for our secondary aim.

### Associations between PSF and PA

**Figure 1.**
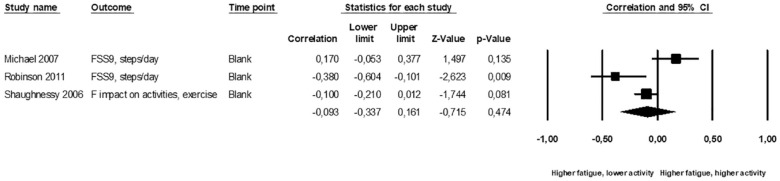
Standardized association between post-stroke fatigue and physical activity. Results are shown for individual studies reporting Pearson’s correlation.

The three studies in the PA meta-analysis included 430 participants.^21,38,41^ PSF was measured with the FSS-9^21,38^ or a single question about the impact of fatigue on daily activities.^41^ PA was measured as steps-per-day^21,38^ and with a question about weekly exercise habits.^41^ The pooled data (Figure 1) showed no correlation between PSF and PA (meta *r* = –0.09; 95% CI = –0.34, 0.16; p = 0.47; I2 = 79%; very low-certainty evidence). Prediction interval was not calculated because of few included studies.

The majority of studies reporting on the association between PSF and PA could not be pooled due to heterogeneity in the statistics used (17 studies, 2621 participants).^23,26–37,39,40,42,43,55^ These studies were synthesized using vote-counting based on direction of association. Table S5 summarizes each study’s statistical methods and association estimates. PSF was most frequently measured using the FSS-7^29,32^ or FSS-9 questionaire.^23,27,30,31,33,39,43^ Eight studies measured PA with activity monitors and reported: step-counts,^23,27,28,32^ time in moderate-vigorous activity,^39,42^ walking time,^33^ or time in activity.^34^ Eight studies used self-reported PA^26,30,31,35–37,40,43^ and one used observational methods^29^ (Table S4). Twelve studies^26,28,30–32,34–37,39,40,43^ found a negative direction of association (i.e. higher PSF-lower PA) and four studies^23,29,33,42^ found a positive association (i.e. higher PSF-higher PA) (Table S6). One study found no direction of association^27^ and was excluded from the synthesis. Seventy-five percent of the studies reported an association between higher PSF and lower PA, but the proportion was not statistically significant (pˆ = 0.75; 95% CI = 0.51, 0.90; p = 0.08; very low-certainty evidence; n = 2566; 16 studies).

Combined, PA meta-analysis and vote-counting showed no association between PSF and reduced PA; very low-certainty evidence.

### Associations between PSF and fitness

**Figure 2.**
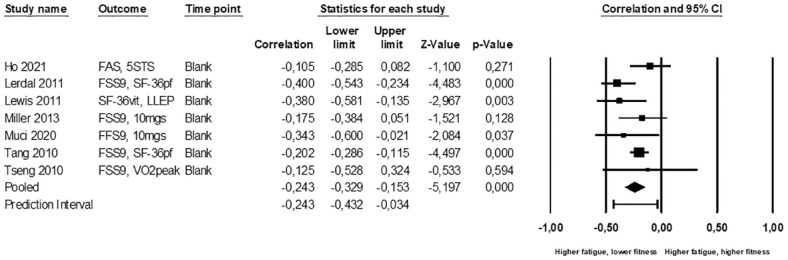
Standardized association between post-stroke fatigue and physical fitness. Results are shown for individual studies reporting Pearson’s correlation.

Seven studies with 878 participants contributed to the meta-analysis of fitness.^46–49,51,52,54^ PSF was measured with the FSS-9,^47,49,51,52,54^ Fatigue Assessment Scale,^46^ or Short Form-36 Vitality subscale.^48^ Fitness was measured by five sit-to-stand (5STS),^46^ Short Form-36 Physical Functioning subscale (SF-36PF),^47,51^ lower-limb extensor-power (LLEP),^48^ 10-meter gait speed (10MGS),^49,54^ or peak oxygen uptake (VO_2_-peak).^52^ We found a statistically significant correlation (Figure 2) between higher PSF and lower fitness (meta *r* = –0.24; 95% CI = –0.33, –0.15; p < 0.001; I2 = 31%; 95% prediction interval = –0.43, –0.03; moderate-certainty evidence).

The remaining seven studies (330 participants) reporting on associations between PSF and fitness could not be pooled because of differing statistics.^21,24,44–46,50,53^ PSF was measured with FSS-9,^21,24,44,45,50,53^ or modified Fatigue Impact Scale.^50^ Fitness was measured with 10MGS,^21,31,45,50^ community ambulation questionnaires,^44,53^ or VO_2_-peak.^24^ Vote-counting showed 5 studies^21,24,44,45,53^ had a negative direction of association (higher PSF-lower fitness) and one study^50^ had a positive direction (higher PSF-higher fitness) (Table S6). One study showed no association^31^ and was excluded from the synthesis. Eighty-three percent of the studies reported an association between higher PSF and lower fitness, but the proportion was not statistically significant (pˆ = 0.83; 95% CI = 0.44, 0.97; p = 0.22, very low-certainty evidence; n = 298; 6 studies).

Combined, the fitness meta-analysis and vote-counting showed a weak association between higher PSF and impaired fitness; low-certainty evidence.

### Risk of bias

For the 30 included studies, the QUIPS domains most frequently assessed as having low risk of bias were “Statistical analysis” (all studies) and “Measurement of prognostic factor, fatigue” (all but three studies).^29,35,41^ The domain most frequently assessed as high risk of bias was “Study confounding” (11 studies)^21,32,33,38,40,41,44,45,49,50,54^ (Table S7). Our GRADE judgments are shown in Tables S8 and S9. The most common reason for downgrading certainty of evidence was risk of bias, followed by inconsistency and imprecision.

## Discussion

To our knowledge, this is the first systematic review including meta-analyses of PSF’s associations with PA and fitness. In contrast to the 2012 review,^5^ there were sufficient studies to pool data and assess certainty of evidence.

Fitness vote-counting showed statistically non-significant, very low-evidence of a higher proportion of studies reporting a negative association between PSF and fitness. The analysis was underpowered^14^ and should be interpreted with care. However, the meta-analysis showed moderate-certainty evidence of a weak association between PSF and impaired fitness. This finding could support the hypothesis of fitness as a protective factor against PSF. Since fitness is a modifiable factor that can be improved by exercise,^6^ exercise training could be a viable intervention for PSF. Zedlitz et al.^56^ reported that cognitive therapy combined with exercise training was better at reducing PSF than cognitive therapy alone and suggested that improving physical endurance may help reduce PSF. Their finding is consistent with studies showing that exercise can reduce fatigue associated with other diagnoses.^7,8^ The potential benefits of exercise training for preventing and treating PSF have been discussed for at least a decade,^57^ but few advances have been made toward this goal. We found no studies designed to investigate the effect of exercise training on PSF. Nonetheless, there is now sufficient evidence of a connection between PSF and fitness to warrant RCTs on exercise interventions to relieve PSF.

We estimated no correlation between PSF and PA in the meta-analysis; very low-certainty evidence. Vote-counting showed very low-certainty evidence of a higher proportion of studies reporting a negative association between PSF and PA, but the test was not statistically significant. Our results are supported by a previous meta-analysis that reported no association between PSF and PA.^58^ In theory, PSF may cause individuals to be less physically active,^5^ but increased PA could also exacerbate PSF.^5^ Causality is difficult to establish and may even vary between individuals. Our findings may reflect such variability. Intrapersonal factors affecting patients’ participation in PA may also play a role: some individuals may choose to avoid PA because of PSF, while others may choose to participate in PA despite PSF.^59^ Because PSF and PA are complex concepts, with many dimensions and associated factors,^4,58,60^ a straightforward causal relationship between the two may not exist. Larger prospective studies, designed to investigate specific hypotheses about PSF’s relationship to PA, are warranted.

In prior meta-analyses, the most frequently-reported risk factors for PSF included female sex, depression, and disability.^60,61^ PA and fitness were not among the factors analyzed, but PA was highlighted as a candidate that could explain more of the variance in PSF.^60^ Based on our meta-analyses, we propose fitness as a more likely candidate. However, the weak association of our estimate suggests there are additional factors involved. Previous research has found a non-linear relationship between PSF and age, with higher PSF among both younger and older individuals.^60,62^ A similar relationship could exist between PSF and fitness, but our study only evaluated linear relationships. Perhaps more likely, age and higher demands on younger individuals^60^ may confound PSF’s relationship with fitness. In our clinical experience, younger patients often suffer from PSF despite being relatively fit. Thus, age-adjusted associations may be stronger, but some included studies did not adjust for age. Well-designed studies are needed to further update the evidence-base on both PSF risk factors and interventions to improve care for stroke survivors of all ages.^63^

### Limitations and strengths

There are several limitations of the included studies. The majority of the evidence came from studies with small sample sizes, using different outcome measures, measured at different time-points. Fatigue instruments are known to measure different dimensions (e.g. intensity, physical fatigue, mental fatigue, impact on activities), and there is a lack of content overlap between instruments.^64^ This means that the fatigue instruments themselves may have varying degrees of associations with PA and fitness. The same applies to the different PA and fitness outcome measures. The included studies also measured associations at different time-points post-stroke, and associations between PSF, PA, and fitness may differ during the acute phase, rehabilitation, or a more stable phase. The extracted associations were reported as a secondary aim or finding in 15 studies,^26,27,30,32,33,38,41,43,44,46,47,49,51–53^ suggesting that these studies were designed and powered for other primary objectives.

As for limitations of our review, since most of the included studies did not have published protocols, we did not use protocols in conjunction with risk of bias assessment as we had planned. This decision did not affect the overall risk of bias in the two studies^23,24^ concerned, nor did it affect the GRADE confidence in certainty of evidence. The PA meta-analysis estimate should be interpreted with caution due to high heterogeneity. A very low certainty of evidence means that the actual estimate of association may differ in either direction. Finally, selection bias may exist, as we only included studies published in English or a Scandinavian language. Our study’s strengths include the multidisciplinary research team, adherence to systematic review guidelines, the extensive search (including TRRs), ability to pool data, and use of GRADE to appraise the certainty of evidence.

### Clinical implications

Given the multiple benefits of PA and exercise for post-stroke recovery,^65^ it is arguable that PA and exercise training should be recommended regardless of whether patients have PSF or not. As PSF can be a barrier to PA,^66^ proper screening, assessment and individually-tailored management of PSF is essential.

## Conclusion

The fitness meta-analysis showed moderate-certainty evidence of an association between PSF and fitness, indicating that being physically fit might protect against PSF. This result needs to be confirmed by RCTs evaluating exercise interventions, with PSF as the primary outcome. The PA meta-analysis showed very low-certainty evidence of no association between PSF and PA, highlighting the lack of large prospective observational studies in this field of research.

## Supplemental Material

sj-docx-1-wso-10.1177_17474930231152132 – Supplemental material for Association of post-stroke fatigue with physical activity and physical fitness: A systematic review and meta-analysis Supplemental material, sj-docx-1-wso-10.1177_17474930231152132 for Association of post-stroke fatigue with physical activity and physical fitness: A systematic review and meta-analysis by Petra Larsson, Julia Bidonde, Unni Olsen, Caryl L Gay, Anners Lerdal, Marie Ursin, Gillian Elizabeth Mead and Elisabeth Edvardsen in International Journal of Stroke
