# Supplementary material for: Association of post-stroke fatigue with physical activity and physical fitness: A systematic review and meta-analysis
Source: Int J Stroke. 2023 Feb 3;18(9):1063–70. doi: 10.1177/17474930231152132 (PMC11044520; doi:10.1177/17474930231152132)
Supplement: sj-docx-1-wso-10.1177_17474930231152132 – Supplemental material for Association of post-stroke fatigue with physical activity and physical fitness: A systematic review and meta-analysis [file sj-docx-1-wso-10.1177_17474930231152132.docx]

**Supplemental material for “Association of post-stroke fatigue with physical activity and physical fitness: a systematic review and meta-analysis”**

By authors Petra Larsson, Julia Bidonde, Unni Olsen, Caryl L Gay, Anners Lerdal, Marie Ursin, Gillian Mead, Elisabeth Edvardsen

| Figure S1 Documentation of the literature search 2 |
| --- |
| Figure S2 Study flow diagram 13 |
| Table S1 Data extraction template 9 |
| Table S2 Prioritization of associations to extract 10 |
| Table S3 Modified Quality In Prognosis Studies (QUIPS) checklist 11 |
| Table S4 Summary statistics across studies 14 |
| Table S5 Study characteristics of individual studies 15 |
| Table S6 Direction of association for fatigue and physical activity/fitness 20 |
| Table S7 Risk of Bias assessment results 21 |
| Table S8 Grading of recommendation, assessment, development and evaluation for meta-analyses and vote-counting analyses separately 22 |
| Table S9 Grading of recommendation, assessment, development and evaluation for meta-analyses and vote-counting analyses combined 22 |

**Figure S1. Documentation of the literature search**

Overview of records identified in each database:

| **Database** | **Records found 22 Aug 2022** | **Records found 30 Nov 2022** |
| --- | --- | --- |
|  |  |  |
| Medline (Ovid): | 792 | 901 |
| Embase (Ovid): | 1709 | 2019 |
| AMED (Ovid): | 75 | 81 |
| CINAHL (Ebsco): | 481 | 530 |
| APA PsycInfo (Ovid): | 227 | 269 |
| ClinicalTrials.gov | 34 (20 with WHO duplicates removed) | 40 (duplicates not removed) |
| WHO ICTRP clinical trials register: | 274 (203 with duplicates removed) | 285 (duplicates not removed |
| Number of records before duplicate check: | 3592 | 4125 |
| **Number of records after duplicate check:** | **2234***  **(2011 from the first 5 databases + 223 trial registry records)** | **2390****  **(2011 + 379 new records from first 5 databases, not including trial registry records)** |

*PL excluded an additional 93 records and added one reference found in a reference list before screening 2298 records in DistillerSR (original search) or Rayyan (updated search)

** We did not check the trial registry records for duplicates after the updated search 30 Nov 2022 as ICTPR records were entered into Rayyan and records from clinicaltrial.gov were screened manually.

All searches were performed 10^th^ – 22^nd^ of August 2021 and 30 November 2022 by Hilde Iren Flaatten, Health information specialist, at the Library of Medicine and Science, University of Oslo.

For information on the selection process, see Study Flow Diagram, page 13 in the supplemental material.

Database: Medline (Ovid) 1946 to August 06, 2021,
 **Ovid MEDLINE(R) ALL 1946 to November 28, 2022**

Search date: 10.08.2021, **30.11.2022**
Number of records: 792, **901**

1 cerebrovascular disorders/ or exp basal ganglia cerebrovascular disease/ or exp brain ischemia/ or exp intracranial arterial diseases/ or exp intracranial arteriovenous malformations/ or exp "intracranial embolism and thrombosis"/ or exp intracranial hemorrhages/ or stroke/ or exp brain infarction/ or stroke, lacunar/ or stroke rehabilitation/

2 (stroke* or poststroke* or cerebrovascular or cerebro vascular or cerebral vascular or cerebralvascular or apoplexia* or transient ischemic attack* or transient ischaemic attack* or hemiplegi* or hemipare* or hemi pare* or hemi plegi*).tw,kf.

3 ((brain* or cerebr* or cerebell* or intracerebral or intra cerebral or intracranial or intra cranial or sub arachnoid* or subarachnoid*) adj3 (ischemi* or ischaemi* or infarct* or thrombo* or emboli* or occlus*)).tw,kf.

4 ((brain* or cerebr* or cerebell* or intracerebral or intra cerebral or intracranial or intra cranial or sub arachnoid* or subarachnoid*) adj3 (haemorrhage* or hemorrhage* or haematoma* or hematoma* or bleed* or aneurysm*)).tw,kf.

5 or/1-4

6 fatigue/ or fatigue syndrome, chronic/ or asthenia/ or mental fatigue/ or muscle fatigue/ or lethargy/

7 (fatig* or astheni* or neurastheni* or tired or tiredness or weary or weariness or ((physical* or mental* or emotion*) adj3 exhaust*) or lassitude or listlessness or letharg* or apath* or malaise).tw,kf.

8 ((low or lack) adj3 energy).tw,kf.

9 or/6-8

10 physical endurance/ or anaerobic threshold/ or metabolic equivalent/ or exercise tolerance/ or physical exertion/ or physical fitness/ or cardiorespiratory fitness/ or oxygen consumption/

11 (fitness or (energy adj2 consumption*) or (energy adj2 metaboli*) or (metaboli* adj2 (threshold* or capacit* or parameter*)) or ((aerobic or anaerobic) adj2 (capacit* or metaboli* or threshold*)) or (energy adj2 expenditure) or (metabolic adj2 equivalent) or endurance or cardiorespiratory or cardio respiratory or cardiopulmonary or cardio pulmonary).tw,kf.

12 ((oxygen or o2) adj2 (consumption* or uptake* or intake* or demand* or requirement* or utilizat* or utilisat*)).tw,kf.

13 (o2consumption* or o2uptake* or o2intake* or o2demand* or o2requirement* or o2utilizat* or o2utilisat* or vo2*).tw,kf.

14 ((physical or motor*) adj2 (condition* or decondition* or function* or exertion* or performance* or factor*)).tw,kf.

15 exp Exercise/ or (exercis* or training or run or running or swim or swimming).tw,kf.

16 Locomotion/ or Motor Activity/

17 ((physical* adj2 activ*) or (physical* adj2 inactiv*) or sedentary or (activit* adj2 (level* or measur* or pattern* or motor* or monitor* or tracking))).tw,kf.

18 Sedentary Behavior/ or walking speed/ or Gait/

19 muscle strength/ or hand strength/ or pinch strength/ or accelerometry/ or actigraphy/

20 ((muscle* adj2 strength*) or (muscle* adj2 power*) or (muscle* adj2 weakness*) or (muscle* adj2 force*)).tw,kf.

21 ((extremit* adj2 strength*) or (extremit* adj2 power*) or (extremit* adj2 weakness*) or (extremit* adj2 force*)).tw,kf.

22 (((hand* or pinch*) adj2 grip*) or ((hand* or pinch*) adj2 strength*) or ((hand* or pinch*) adj2 power*) or ((hand* or pinch*) adj2 force*) or ((hand* or pinch*) adj2 weakness*)).tw,kf. (12013)

23 (acceleromet* or actigraph* or actimetr* or step count* or pedomet* or steps per day or walk or walking or gait*).tw,kf.

24 ((step adj2 test*) or (strength* adj2 (measur* or test*)) or (leg adj2 press) or (stand* adj2 test*) or (stair climb* adj2 test*)).tw,kf.

25 or/10-24

26 5 and 9 and 25

27 (Animal Experimentation/ or exp Animals/ or exp Models, Animal/) not Humans/ (4758126)

28 ((veterinar* or animal or animals or rabbit or rabbits or rodent or rodents or rat or rats or mouse or mice or hamster or hamsters or pig or pigs or piglet or piglets or porcine or pigeon* or horse* or equine or cow or cows or bovine or goat or goats or sheep or lamb or lambs or monkey or monkeys or murine or ovine or dog or dogs or canine or cat or cats or feline or dolphin*) not (patient or patients or human or humans)).ti.

29 26 not (27 or 28)

30 (power stroke* or stroke volume* or stroke rate* or (strokes adj2 minute*)).tw,kf.

31 29 not 30

32 limit 31 to (danish or english or norwegian or swedish)

Database: Embase Classic+Embase 1947 to 2021 August 06,
 **Embase Classic+Embase 1947 to 2022 November 28**

Search date: 10.08.2021, **30.11.2022**
Number of records: 1709, **2019**

1 cerebrovascular disease/ or exp basal ganglion hemorrhage/ or exp brain hemangioma/ or exp brain hematoma/ or exp brain hemorrhage/ or exp brain infarction/ or exp brain ischemia/ or cerebral artery disease/ or exp cerebrovascular accident/ or exp cerebrovascular malformation/ or exp intracranial aneurysm/ or exp occlusive cerebrovascular disease/ or stroke rehabilitation/

2 (stroke* or poststroke* or cerebrovascular or cerebro vascular or cerebral vascular or apoplexia* or transient ischemic attack* or transient ischaemic attack* or hemiplegi* or hemipare* or hemi pare* or hemi plegi*).tw,kw.

3 ((brain* or cerebr* or cerebell* or intracerebral or intra cerebral or intracranial or intra cranial or sub arachnoid* or subarachnoid*) adj3 (ischemi* or ischaemi* or infarct* or thrombo* or emboli* or occlus* or aneurysm*)).tw,kw.

4 ((brain* or cerebr* or cerebell* or intracerebral or intra cerebral or intracranial or intra cranial or sub arachnoid* or subarachnoid*) adj3 (haemorrhage* or hemorrhage* or haematoma* or hematoma* or bleed*)).tw,kw.

5 or/1-4

6 fatigue/ or chronic fatigue syndrome/ or exhaustion/ or lassitude/ or muscle fatigue/ or lethargy/ or asthenia/

7 (fatig* or astheni* or neurastheni* or tired or tiredness or weary or weariness or ((physical* or mental* or emotion*) adj3 exhaust*) or lassitude or listlessness or letharg* or apath* or malaise).tw,kw.

8 ((low or lack) adj3 energy).tw,kw.

9 or/6-8

10 endurance/ or "physical activity, capacity and performance"/ or anaerobic threshold/ or oxygen consumption/ or aerobic capacity/ or metabolic capacity/ or metabolic equivalent/ or metabolic parameters/ or exercise tolerance/ or physical capacity/ or physical tolerance/ or fitness/ or cardiorespiratory fitness/ or cardiovascular parameters/ or respiratory tract parameters/

11 (fitness or (energy adj2 consumption*) or (energy adj2 metaboli*) or (metaboli* adj2 (threshold* or capacit* or parameter*)) or ((aerobic or anaerobic) adj2 (capacit* or metaboli* or threshold*)) or (energy adj2 expenditure) or (metabolic adj2 equivalent) or endurance or cardiorespiratory or cardio respiratory or cardiopulmonary or cardio pulmonary).tw,kw.

12 ((oxygen or o2) adj2 (consumption* or uptake* or intake* or demand* or requirement* or utilizat* or utilisat*)).tw,kw.

13 (o2consumption* or o2uptake* or o2intake* or o2demand* or o2requirement* or o2utilizat* or o2utilisat* or vo2*).tw,kw.

14 ((physical or motor*) adj2 (condition* or decondition* or function* or exertion* or performance* or factor*)).tw,kw.

15 exp Exercise/ or (exercis* or training or run or running or swim or swimming).tw,kw.

16 exp locomotion/ or motor activity/

17 ((physical* adj2 activ*) or (physical* adj2 inactiv*) or sedentary or (activit* adj2 (level* or measur* or pattern* or motor* or monitor* or tracking))).tw,kw.

18 sedentary lifestyle/ or walking speed/ or gait/

19 muscle strength/ or hand strength/ or grip strength/ or pinch strength/ or accelerometry/ or accelerometer/ or actimetry/

20 ((muscle* adj2 strength*) or (muscle* adj2 power*) or (muscle* adj2 weakness*) or (muscle* adj2 force*)).tw,kw.

21 ((extremit* adj2 strength*) or (extremit* adj2 force*) or (extremit* adj2 power*) or (extremit* adj2 weakness*)).tw,kw.

22 (((hand* or pinch*) adj2 grip*) or ((hand* or pinch*) adj2 force*) or ((hand* or pinch*) adj2 strength*) or ((hand* or pinch*) adj2 power*) or ((hand* or pinch*) adj2 weakness*)).tw,kw. (17564)

23 (acceleromet* or actigraph* or actimetr* or step count* or pedomet* or steps per day or walk or walking or gait*).tw,kw.

24 ((step adj2 test*) or (strength* adj2 (measur* or test*)) or (leg adj2 press) or (stand* adj2 test*) or (stair climb* adj2 test*)).tw,kw.

25 or/10-24

26 5 and 9 and 25

27 (power stroke* or stroke volume* or stroke rate* or (strokes adj2 minute*)).tw,kw.

28 26 not 27

29 (exp animal/ or exp animal model/ or nonhuman/) not exp human/

30 28 not 29

31 limit 30 to conference abstracts

32 30 not 31

33 limit 32 to (danish or english or norwegian or swedish)

Database: AMED (Allied and Complementary Medicine) 1985 to August 2021
 **AMED (Allied and Complementary Medicine) 1985 to November 2022**

Search date: 10.08.2021, **30.11.2022**Number of records: 75, **81**

1 (stroke* or poststroke* or cerebrovascular or cerebro vascular or cerebral vascular or apoplexia* or transient ischemic attack* or transient ischaemic attack* or hemiplegi* or hemipare* or hemi pare* or hemi plegi*).mp.

2 ((brain* or cerebr* or cerebell* or intracerebral or intra cerebral or intracranial or intra cranial or sub-arachnoid* or subarachnoid*) adj3 (ischemi* or ischaemi* or infarct* or thrombo* or emboli* or occlus*)).mp.

3 ((brain* or cerebr* or cerebell* or intracerebral or intra cerebral or intracranial or intra cranial or sub-arachnoid* or subarachnoid*) adj3 (haemorrhage* or hemorrhage* or haematoma* or hematoma* or bleed* or aneurysm*)).mp.

4 or/1-3

5 (fatig* or astheni* or neurastheni* or tired or tiredness or weary or weariness or ((physical* or mental* or emotion*) adj3 exhaust*) or lassitude or listlessness or letharg* or apath* or malaise).mp.

6 ((low or lack) adj3 energy).mp.

7 or/5-6

8 (fitness or (energy adj2 consumption*) or (energy adj2 metaboli*) or (metaboli* adj2 (threshold* or capacit* or parameter*)) or ((aerobic or anaerobic) adj2 (capacit* or metaboli* or threshold*)) or (energy adj2 expenditure) or (metabolic adj2 equivalent) or endurance or cardiorespiratory or cardio respiratory or cardiopulmonary or cardio pulmonary).mp.

9 ((oxygen or o2) adj2 (consumption* or uptake* or intake* or demand* or requirement* or utilizat* or utilisat*)).mp.

10 (o2consumption* or o2uptake* or o2intake* or o2demand* or o2requirement* or o2utilizat* or o2utilisat* or vo2*).mp.

11 ((physical or motor*) adj2 (condition* or decondition* or function* or exertion* or performance* or factor*)).mp.

12 (exercis* or training or run or running or swim or swimming).mp.

13 ((physical* adj2 activ*) or (physical* adj2 inactiv*) or sedentary or (activit* adj2 (level* or measur* or pattern* or motor* or monitor* or tracking))).mp.

14 ((muscle* adj2 strength*) or (muscle* adj2 power*) or (muscle* adj2 weakness*) or (muscle* adj2 force*)).mp.

15 ((extremit* adj2 strength*) or (extremit* adj2 power*) or (extremit* adj2 weakness*) or (extremit* adj2 force*)).mp.

16 (((hand* or pinch*) adj2 grip*) or ((hand* or pinch*) adj2 strength*) or ((hand* or pinch*) adj2 power*) or ((hand* or pinch*) adj2 force*) or ((hand* or pinch*) adj2 weakness*)).mp.

17 (acceleromet* or actigraph* or actimetr* or step count* or pedomet* or steps per day or walk or walking or gait*).mp.

18 ((step adj2 test*) or (strength* adj2 (measur* or test*)) or (leg adj2 press) or (stand* adj2 test*) or (stair climb* adj2 test*)).mp.

19 or/8-18

20 4 and 7 and 19

21 (power stroke* or stroke volume* or stroke rate* or (strokes adj2 minute*)).mp.

22 20 not 21

23 limit 22 to (danish or english or norwegian or swedish)

Database: APA PsycInfo 1806 to August Week 1 2021
 **APA PsycInfo 1806 to November Week 3 2022**Search date: 10.08.2021, **30.11.2022**
Number of records identified: 227, **269**

1 (stroke* or poststroke* or cerebrovascular or cerebro vascular or cerebral vascular or apoplexia* or transient ischemic attack* or transient ischaemic attack* or hemiplegi* or hemipare* or hemi pare* or hemi plegi*).mp.

2 ((brain* or cerebr* or cerebell* or intracerebral or intra cerebral or intracranial or intra cranial or sub arachnoid* or subarachnoid*) adj3 (ischemi* or ischaemi* or infarct* or thrombo* or emboli* or occlus*)).mp.

3 ((brain* or cerebr* or cerebell* or intracerebral or intra cerebral or intracranial or intra cranial or sub arachnoid* or subarachnoid*) adj3 (haemorrhage* or hemorrhage* or haematoma* or hematoma* or bleed* or aneurysm*)).mp.

4 or/1-3

5 (fatig* or astheni* or neurastheni* or tired or tiredness or weary or weariness or ((physical* or mental* or emotion*) adj3 exhaust*) or lassitude or listlessness or letharg* or apath* or malaise).mp.

6 ((low or lack) adj3 energy).mp.

7 or/5-6

8 (fitness or (energy adj2 consumption*) or (energy adj2 metaboli*) or (metaboli* adj2 (threshold* or capacit* or parameter*)) or ((aerobic or anaerobic) adj2 (capacit* or metaboli* or threshold*)) or (energy adj2 expenditure) or (metabolic adj2 equivalent) or endurance or cardiorespiratory or cardio respiratory or cardiopulmonary or cardio pulmonary).mp.

9 ((oxygen or o2) adj2 (consumption* or uptake* or intake* or demand* or requirement* or utilizat* or utilisat*)).mp.

10 (o2consumption* or o2uptake* or o2intake* or o2demand* or o2requirement* or o2utilizat* or o2utilisat* or vo2*).mp.

11 ((physical or motor*) adj2 (condition* or decondition* or function* or exertion* or performance* or factor*)).mp.

12 (exercis* or training or run or running or swim or swimming).mp.

13 ((physical* adj2 activ*) or (physical* adj2 inactiv*) or sedentary or (activit* adj2 (level* or measur* or pattern* or motor* or monitor* or tracking))).mp.

14 ((muscle* adj2 strength*) or (muscle* adj2 power*) or (muscle* adj2 weakness*) or (muscle* adj2 force*)).mp.

15 ((extremit* adj2 strength*) or (extremit* adj2 power*) or (extremit* adj2 weakness*) or (extremit* adj2 force*)).mp.

16 (((hand* or pinch*) adj2 grip*) or ((hand* or pinch*) adj2 strength*) or ((hand* or pinch*) adj2 power*) or ((hand* or pinch*) adj2 force*) or ((hand* or pinch*) adj2 weakness*)).mp.

17 (acceleromet* or actigraph* or actimetr* or step count* or pedomet* or steps per day or walk or walking or gait*).mp.

18 ((step adj2 test*) or (strength* adj2 (measur* or test*)) or (leg adj2 press) or (stand* adj2 test*) or (stair climb* adj2 test*)).mp.

19 or/8-18

20 4 and 7 and 19

21 (power stroke* or stroke volume* or stroke rate* or (strokes adj2 minute*)).mp.

22 20 not 21

23 limit 22 to (danish or english or norwegian or swedish)

24 limit 23 to "0200 book"

25 23 not 24

Database: **CINAHL (Ebsco)**

Search date: 10.08.2021, **30.11.2022**
Number of records: 481, **530**

S1 (MH "Basal Ganglia Cerebrovascular Disease+") OR (MH "Cerebrovascular Disorders") OR (MH "Carotid Artery Diseases+") OR (MH "Cerebral Ischemia+") OR (MH "Hypoxia-Ischemia, Brain+") OR (MH "Stroke, Lacunar") OR (MH "Cerebral Small Vessel Diseases") OR (MH "Intracranial Arterial Diseases+") OR (MH "Intracranial Embolism and Thrombosis+") OR (MH "Intracranial Thrombosis+") OR (MH "Intracranial Hemorrhage+") OR (MH "Stroke+") OR (MH "Stroke Patients")

S2 (stroke* OR poststroke* OR cerebrovascular OR “cerebro vascular” OR “cerebral vascular” OR apoplexia* OR “transient ischemic attack*” OR “transient ischaemic attack*” OR hemiplegi* OR hemipare* OR "hemi plegi*" OR "hemi pare*")

S3 ((brain* OR cerebr* OR cerebell* OR intracerebral OR “intra cerebral” OR intracranial OR “intra cranial” OR “sub arachnoid*” OR subarachnoid*) N2 (ischemi* OR ischaemi* OR infarct* OR thrombo* OR emboli* OR occlus* OR haemorrhage* OR hemorrhage* OR haematoma* OR hematoma* OR bleed*))

S4 S1 OR S2 OR S3

S5 (MH "Asthenia") OR (MH "Fatigue") OR (MH "Fatigue Syndrome, Chronic") OR (MH "Mental Fatigue") OR (MH "Muscle Fatigue")

S6 (fatig* OR astheni* OR neurastheni* OR tired OR tiredness OR weary OR weariness OR ((physical* OR mental* OR emotion*) N2 exhaust*) OR lassitude OR listlessness OR letharg* OR apath* OR malaise OR ((low OR lack) N2 energy))

S7 S5 OR S6

S8 (MH "Physical Endurance+") OR (MH "Exercise Tolerance+") OR (MH "Aerobic Capacity") OR (MH "Exertion") OR (MH "Anaerobic Threshold") OR (MH "Exercise+") OR (MH "Exercise Intensity") OR (MH "Exercise Physiology+") OR MH ("Physical Fitness") OR (MH "Cardiorespiratory Fitness") OR (MH "Oxygen Consumption+")

S9 (fitness OR (energy N1 consumption*) OR (energy N1 metaboli*) OR (metaboli* N1 (threshold* OR capacit* OR parameter*)) OR ((aerobic OR anaerobic) N1 (capacit* OR metaboli* OR threshold*)) OR (energy N1 expenditure) OR (metabolic N1 equivalent) OR endurance OR cardiorespiratory OR “cardio respiratory” OR cardiopulmonary OR “cardio pulmonary”)

S10 (((oxygen OR o2) N1 (consumption* OR uptake* OR intake* OR demand* OR requirement* OR utilizat* OR utilisat*)) ) OR ( (o2consumption* OR o2uptake* OR o2intake* OR o2demand* OR o2requirement* OR o2utilizat* OR o2utilisat* OR vo2*) ) OR ( ((physical OR motor*) N1 (condition* OR decondition* OR function* OR exertion* OR performance* OR factor*)) )

S11 (exercis* OR training OR run OR running OR swim OR swimming)

S12 ((physical* N1 activ*) OR (physical* N1 inactiv*) OR sedentary OR (activit* N1 (level* OR measur* OR pattern* OR motor* OR monitor* OR tracking)))

S13 (MH "Life Style, Sedentary") OR (MH "Locomotion+") OR (MH "Motor Activity+") OR (MH "Muscle Strength+") OR (MH "Muscle Strengthening+") OR (MH "Therapeutic Exercise") OR (MH "Actigraphy") OR (MH "Accelerometry")

S14 ((muscle* N1 strength*) OR (muscle* N1 power*) OR (muscle* N1 weakness*) OR (muscle* N1 force*))

S15 ((extremit* N1 strength*) OR (extremit* N1 power*) OR (extremit* N1 weakness*) OR (extremit* N1 force*))

S16 (((hand* OR pinch*) N1 grip*) OR ((hand* OR pinch*) N1 strength*) OR ((hand* OR pinch*) N1 power*) OR ((hand* OR pinch*) N1 force*) OR ((hand* OR pinch*) N1 weakness*))

S17 (acceleromet* OR actigraph* OR actimetr* OR step count* OR pedomet* OR “steps per day” OR walk OR walking OR gait*)

S18 (step N1 test*) N1 (strength* N1 (measur* OR test*)) OR (leg N1 press) OR (stand* N1 test*) OR (stair climb* N1 test*)

S19 S8 OR S9 OR S10 OR S11 OR S12 OR S13 OR S14 OR S15 OR S16 OR S17 OR S18

S20 S4 AND S7 AND S19

S21 “power stroke” OR “power strokes” OR “stroke volume” OR “stroke volumes” OR “stroke rate” OR “stroke rates” OR (strokes N1 minute*)

S22 S20 NOT S21 Limiters - Language: Danish, English, Norwegian, Swedish

**Database:** ClinicalTrials.gov
**Search date:** 22.08.2021, **30.11.2022
Number of records identified:** 34, **40**

Observational Studies | fatigue AND (stroke OR strokes OR poststroke OR poststrokes OR cerebral OR brain OR hemorrhage OR haemorrhage OR hemiplegia)
[Search of: Observational Studies | fatigue AND (stroke OR strokes OR poststroke OR poststrokes OR cerebral OR brain OR hemorrhage OR haemorrhage OR hemiplegia) - List Results - ClinicalTrials.gov](https://www.clinicaltrials.gov/ct2/results?cond=fatigue+AND+%28stroke+OR+strokes+OR+poststroke+OR+poststrokes+OR+cerebral+OR+brain+OR+hemorrhage+OR+haemorrhage+OR+hemiplegia%29&term=&type=Obsr&rslt=&age_v=&gndr=&intr=&titles=&outc=&spons=&lead=&id=&cntry=&state=&city=&dist=&locn=&rsub=&strd_s=&strd_e=&prcd_s=&prcd_e=&sfpd_s=&sfpd_e=&rfpd_s=&rfpd_e=&lupd_s=&lupd_e=&sort=)

**Database:** [International Clinical Trials Registry Platform](https://trialsearch.who.int/AdvSearch.aspx)

[ICTRP Search Portal Advanced Search (who.int)](https://trialsearch.who.int/AdvSearch.aspx)

**Search date:** 22.08.2021, **30.11.2022**
**Number of records identified:** 274

Søk 1:

(stroke* OR poststroke* OR cerebr* OR brain* OR hemorrhage* OR haemorrhage* OR hemiplegia*) AND (fatigue OR fitness) - in title – Recruitment status: ALL (213 Trials)

**(226 records for 225 trials found)**

Søk 2:

(stroke* OR poststroke* OR cerebr* OR brain* OR hemorrhage* OR haemorrhage OR hemiplegia*) AND (fatigue OR fitness) – in condition - Recruitment status: ALL (61 Trials)

**(59 records for 55 trials found)**

**Table S1. Data extraction template**

| **Data type** | **Extracted data items** |
| --- | --- |
| Publication details | First author, publication year, country |
| Study characteristics | Study design, study and recruitment setting, inclusion/exclusion criteria, aim, length of follow-up, sample size, sample size for association |
| Patient characteristics | Age, sex, stroke type and location, time since stroke, functional impairments after stroke, fatigue at baseline, physical activity and/or physical fitness level at baseline |
| Fatigue outcome | Fatigue outcome measure and cut-off used, if relevant |
| Physical activity, physical fitness outcome | Physical activity and physical fitness measures, including unit of measurement |
| Measure of association | Point estimate (i.e. numerical result), precision (e.g. 95% CI, SE), p-value, and statistical method used (e.g. Pearson correlation, linear regression standardized beta, multiple logistic regression) |

**Table S2. Prioritization of associations to extract**

| **Author** | **Association outcome measure** | **Reason for inclusion/ comment** |
| --- | --- | --- |
| Duncan 2015^1^ | Steps per day | 5. Steps per day was more frequently reported  6. Twelve months was the time point most similar to other studies |
|  | ~~Time per day stepping~~ |  |
|  | ~~Time per day in up-right position~~ |  |
| Fini 2021^2^ | Steps per day | 5. Steps per day was more frequently reported |
|  | ~~Moderate to vigorous PA~~ |  |
| Hamre 2021^3^ | ~~Self-reported PA, univariate~~ | 2. Multivariate analysis over univariate  6. Twelve months was the time point most similar to other studies |
|  | ~~Self-reported PA, uni and multivariate acute phase and 3 months~~ |  |
|  | Self-reported PA, multivariate at 12 months |  |
| Ho 2021^4^ | 5 sit-to-stand, Pearson correlation | 1. Pearson correlation, used in meta-analysis. (Both associations were statistically non-significant). |
|  | ~~5 sit-to-stand, multivariate~~ |  |
| Hoang 2012^5^ | Gait speed | 5. Steps per day was more frequently reported |
|  | ~~6-minute walk test~~ |  |
| Kirchberger 2022^6^ | ~~Baseline PA to 3 month fatigue~~ | 6. More similar to other timepoints/ time  since stroke |
|  | Baseline PA to 12 month fatigue |  |
| Lerdal 2011^7^ | Short Form-36 physical function (Pearson correlation) | 1. Pearson correlation, used in meta-analysis. (Both associations were statistically significant). |
|  | ~~Short Form-36 Physical function (multiple)~~ |  |
| Lewis 2011^8^ | Lower limb extensor power, Pearson correlation | 1. Pearson correlation (Pearson correlation and 3 out of 4 multivariate analyses were statistically significant) |
|  | ~~Lower limb extensor power in multivariate analyses~~ |  |
| Mahendran 2020^9^ | Steps per day Baseline to 6 month fatigue | 6. More similar to other timepoints/ time since stroke |
|  | ~~Steps per day Baseline to 1 and 3 month fatigue~~ |  |
| Michael 2007^10^ | Steps per day | 5. Steps per day was more frequently reported |
|  | ~~Low intensity stepping/medium intensity stepping/ high intensity stepping~~ |  |
|  | Gait speed | 5. Gait speed was more frequently reported |
|  | ~~VO2 peak~~ |  |
| Miller 2013^11^ | Gait speed | 5. Gait speed was more frequently reported |
|  | ~~6-minute walk test~~ |  |
| Oyake 2021^12^ | VO2 peak | 5. VO2 peak was more frequently reported |
|  | ~~VO2 at ventilator threshold~~ |  |
| Pedersen 2022^13^ | ~~Low PA baseline to 7 year fatigue, OR~~ | 6. More similar to other timepoints/ time since stroke |
|  | ~~Low PA baseline to 7 year fatigue, Spearman correlation~~ |  |
|  | Low PA at 7 years |  |
| Robinson 2011^14^ | Steps per day | 3. Steps per day was valid  4. Steps per day was objective  5. Steps per day was more frequently reported |
|  | ~~Walking related activity (WRA)~~ |  |
| Sánchez-Sánchez 2021^15^ | ~~Light PA~~ | 2. Multivariate association |
|  | ~~Moderate-to vigorous PA (Spearman correlation)~~ |  |
|  | Moderate-to-vigorous PA (multivariate) |  |
| Schüler 2021^16^ | Self-reported PA | 6. More frequently reported (and self-reported Sport activity had very low “n”) |
|  | ~~Self-reported Sport activity~~ |  |

PA=physical activity, VO_2_ peak=peak oxygen uptake

Note: Crossed-out association outcome measures indicate associations that were not prioritized/extracted

Prioritizing order: 1. Statistical method (Pearson correlation) to maximize the number of studies in the meta-analyses; 2. Statistical method- multivariate analyses over univariate; 3. Valid measures; 4. Objective measures; 5. Similarity to other outcome measures; 6. Similarity to other included associations (time-point, time since stroke)

**Table S3. Modified Quality In Prognosis Studies (QUIPS) checklist^17^**

| **Domains** | **Prompting items (customized)** | **Ratings** |
| --- | --- | --- |
| **1. Study participation**  Goal: To judge the risk of selection bias (likelihood that relationship between PF and outcome is different for participants and eligible non-participants). | a) Adequate description of sampling frame and recruitment. Minimum: information available on the patient's recruitment (from which health service) and how data collection was performed.  b) Period of recruitment (month and year)  c) Setting and geographic location of recruitment/name of hospital.  d) Adequate description of inclusion/exclusion criteria. At least one inclusion and one exclusion criteria should be given.  e) Adequate participation of eligible individuals, at least 67%.  f) Baseline sample adequately described for key characteristics. At least basic information on gender, age, together with stroke-related information such as time since stroke/stroke type/stroke location/functional outcome/fatigue. | High risk of bias: The association between fatigue and physical activity or physical fitness is very likely to be different for eligible participants and eligible non-participants.  Moderate risk of bias: The association between fatigue and physical activity or physical fitness may be different for eligible participants and eligible non-participants.  Low risk of bias: The association between fatigue and physical activity or physical fitness is unlikely to be different for eligible participants and eligible non-participants. |
| **2. Study attrition** (not assessed for cross-sectional studies)  Goal: To judge the risk of attrition bias (likelihood that relationship between PF and outcome are different for completing and non-completing participants). | a) Adequate response rate. Proportion of sample completing the study and providing outcome data, at least 67%.  b) Description of attempts to collect information on drop-outs (methods and timing).  c) Reasons for loss to follow up provided  d) Adequate description of participants lost to follow up (e.g. gender, age, stroke-related information).  e) No important differences between key characteristics and outcomes in participants who completed the study and those who did not. | High risk of bias: The association between fatigue and physical activity or physical fitness is very likely to be different for completing and non-completing participants.  Moderate risk of bias: The association between fatigue and physical activity or physical fitness may be different for completing and non-completing participants. Low risk of bias: The association between fatigue and physical activity or physical fitness is unlikely to be different for completing and non-completing participants. |
| **3. Prognostic factor measurement**  Goal: To judge the risk of measurement bias related to how fatigue was measured. | a) A clear description of fatigue is provided.  b) Method of measuring fatigue is valid and reliable and at least one reference of reliability/validity study given.  c) Continuous variables are reported or appropriate cut-points (i.e. not data-dependent) is used  d) Adequate proportion of the sample has complete fatigue data, at least 67%.  e) Appropriate imputation for missing fatigue data. | High risk of bias: The fatigue measurement is very likely to be biased because of measurement methods.  Moderate risk of bias: The fatigue measurement may be biased because of measurement methods. Low risk of bias: The fatigue measurement is unlikely to be biased because of measurement methods. |
| **4. Outcome measurement**  Goal: To judge the risk of bias related to the measurement of outcome. | a) A clear description of physical activity or physical fitness is provided.  b) Method of measuring physical activity or physical fitness is valid for the stroke population and reference is given.  c) Measurement method is the same for all study participants and measurement setting is comparable.  d) Adequate proportion of the sample has complete fatigue data, at least 67%. | High risk of bias: The physical activity or physical fitness measurement is very likely to be biased because of measurement methods.  Moderate risk of bias: The physical activity or physical fitness measurement may be biased because of measurement methods.  Low risk of bias: The physical activity or physical fitness measurement is unlikely to be biased because of measurement methods. |
| **5. Study confounding**  Goal: To judge the risk of bias due to confounding. | a) At least one confounder is taken measured.  b) Adequate description of confounder, e.g. measurement methods used, data collection procedure.  c) The confounder measurement/s is/are validated/reliable in stroke and described and referenced in the paper.  d) Confounder measurement methods are the same for all participants.  e) Adequate proportion of sample with confounder data, at least 67% %, or if not- imputation is used.  f) Important potential confounders are accounted for in the study design, or confounders are described as such and included in the analysis. | High risk of bias: The observed association between fatigue and physical activity or physical fitness is very likely to be confounded by another factor.  Moderate risk of bias: The observed association between fatigue and physical activity or physical fitness may be confounded by another factor.  Low risk of bias: The observed association between fatigue and physical activity or physical fitness unlikely to be confounded by another factor. |
| **6. Statistical analysis and reporting**  Goal: To judge the risk of bias related to the statistical analysis and presentation of results. | a) Sufficient presentation of data to assess the adequacy of the analysis.  b) Adequate statistical model for the study design and some form of description of statistical analyses available.  c) No selective reporting, e.g. analyses of fatigue and physical activity and physical fitness described in the methods sections should be included in results. | High risk of bias: The reported results are very likely to be biased related to analysis or reporting  Moderate risk of bias: The reported results may be biased related to analysis or reporting  Low risk of bias: The reported results are unlikely to be biased related to analysis or reporting |

**Supplementary Figure 2. Study flow diagram**

**Identification of studies via databases and registers**

**Records identified from databases and trial registry records (n=4125)**

- Medline (Ovid): 901
- Embase (Ovid): 2019
- AMED (Ovid): 81
- CINAHL (Ebsco): 530
- APA PsycInfo (Ovid): 269
- ICTRP: 285
- ClinicalTrials.gov: 40

Duplicates removed from the first 5 databases*

(n=1503 articles)

**Identification**

Additional records found through manual search of reference lists (n=1)

Records excluded (n=2146)

Records screened by title and abstract (n=2298)**

**Full-text articles excluded (n=118)**

- Ineligible study design (n=32)
- Ineligible fatigue outcome (n = 26)
- Ineligible physical fitness/activity outcomes (n=21)
- Association not reported (n=26)
- Direction of association not reported (n=1)
- Study protocols (n=3)
- Not peer-reviewed full-text (n=3)
- Age <18 years (n=2)
- Not in English or Scandinavian language (n=1)
- Not stroke (n=1)
- Companion paper with same outcome (n=1)
- Duplicates (n=1)

**Trial registry records excluded (n=323)**

**Screening**

Full-text articles assessed for eligibility (n=152)

Trial registry records from ICTRP or ClinicalTrials.gov (n=325)

**Full-text articles included (n=34):**

- Unique studies (n=32)
- Companion papers (n=2): one published protocol for a completed study and one second publication from same study

**Trial registry records (n=2)**, one ongoing, one completed with study included

**Included**

* Duplicate check for the trial registry records was only performed after the search in August 2021 (308 records found and 85 excluded). For the updated search of Nov 2022 PL screened all the ICTRP trial registry records in Rayyan, while clinicaltrial.gov records, registered after 22 August 2021, were screened manually. No new eligible records were found.

**Not including trial registry records. They were screened manually at full-text level because of technical difficulties.

Note: Two of the three study protocols (i.e. published protocols, trial registry records) that met our inclusion criteria described included studies,^2,12^ and the third study was ongoing.^20^ One study was excluded following unsuccessful attempts to obtain missing data^21^ and one companion paper was excluded because an association for physical fitness, from the same sample, was already included.^22^

**Table S4. Summary statistics across studies, n=32 studies.**

| **Data point** | Number of studies (unless otherwise stated) |
| --- | --- |
| **Total number of participants** | Participants: n=4721  Males: n=2604 (55%) |
| **Median of the mean age across studies, n=26*** | 63 years, range 54-75 years |
| **Study design** | Cross-sectional: 23 |
|  | Longitudinal prospective observational: 7** |
|  | Randomized controlled trials : 2*** |
| **Continent of origin** | Africa: 1 |
|  | Asia: 7 |
|  | Europe: 14 |
|  | North America: 6 |
|  | Oceania: 4 |
| **Time since stroke at recruitment** | 0-1 months: 3 |
|  | >1-3 months: 2 |
|  | >3-6 months: 1 |
|  | >12 months: 4 |
|  | Mixed time since stroke: 22 |
| **Stroke type** | Hemorrhagic and ischemic: 18 |
|  | Ischemic: 5 |
|  | Not reported: 9 |
| **Fatigue outcome measure** | Fatigue assessment scale: 5 |
|  | Fatigue severity scale - 9 items: 19 |
|  | Fatigue severity scale -7 items: 2 |
|  | Fatigue scale for motor and cognitive functions: 1 |
|  | Daily fatigue impact scale:1 |
|  | Modified Fatigue impact scale: 3 |
|  | Short Form-36 Vitality: 1 |
| **Physical fitness outcomes** | 10-meter gait speed: 6 |
|  | Peak oxygen uptake, VO2peak: 2 |
|  | 5 sit-to-stand (5STS): 1 |
|  | Lower limb extensor power: 1 |
|  | Short Form-36 which sub-scale? Otherwise QoL : 2 |
|  | Community ambulation questionnaire: 2 |
| **Physical activity outcomes** | Steps/day: 6 |
|  | Moderate to vigorous activity: 2 |
|  | Total physical activity time: 1 |
|  | Low physical activity: 2 |
|  | % of daytime in upright position (behavioral mapping): 1 |
|  | International Physical Activity Questionnaire: 1 |
|  | Self-reported physical activity/exercise: 5 |

* Two studies reported only median age

** Two studies reported cross-sectional data from 12 months, one study reported cross-sectional data from 7 years, four studies reported data on associations between baseline and later time points

***Cross-sectional data from baseline

**Table S5. Study characteristics of individual studies**

| **Author (year)** | **Country** | **Study design** | **Sample size (no of patients analyzed if different)** | **Gender (M/F)** | **Baseline mean age (years)** | **Mean time since stroke** | **Type of stroke** | **Fatigue measure, mean baseline score,**  **cases%** | **Outcome measure, mean baseline value/score** | **Analysis** |
| --- | --- | --- | --- | --- | --- | --- | --- | --- | --- | --- |
| Almhdawi^23^  (2021) | Jordan | CS | 153 | 88/65 | 56 | Mean 27.37 (SD 44.11) mo | H+I | MFIS,  Mean=45.32 (SD=17.67),  69.9% | Self-reported weekly sports hours, 1.31 (SD 2.70) | Multiple linear regression, β=−0.94 [95%CI= −1.73, −0.14], p=0.022 |
| Danks^24^  (2017) | USA | CS | 55 | 33/22 | 54 | Median 47, range 4-366  mo | NR | FSS-9 (sum)**,  Mean=34 (SD 14),  NR | StepWatch Activity Monitor (SAM), Mean 5816, SD 3293, Steps/d | Multiple linear regression, β=0.00, p=1.0 |
| Duncan^1^  (2015) | UK | P | 136(84) | 58/78 | 72, median | 12 mo | H+I | FAS at 12 mo,  Median=22 (IQR=17-22),  20% | ActivPal,  Median 4.314, IQR 1.657–6.890,  Steps/d *at 12 mo* | Spearman correlation,  rho=-0.35, p=0.007 |
| Durcan^25^  (2016) | Ireland | CS | 40 | 22/18 | 66 | Range 1-3 y | H+I | FSS-9,  NR,  NR | Community ambulation questionnaire,  Community walkers n=23 | Bivariate logistic regression, OR=0.98, p=0.38 |
| Egerton^26^  (2015) | Norway | P | 257(199) | 138/119 | 75 | Baseline <14 d and 3 mo (two time points) | H+I | FSS-7,  NR,  34.6% | % of daytime spent upright,  Mean 10.9, SD 9.2 | Multiple logistic regression, OR=0.03, [95%CI= 0.98, 1.07], p=0.58 |
| Fini^2^  (2021) | Australia | P | 79(68) | 54/25 | 65 | Baseline after rehab and 24 mo (two time points) | H+! | FSS-9,  NR,  NR | SenseWear MF Armband, Median 3685, IQR 638–6596 Steps/d | Multiple linear regression, mean difference (effect size) =4, [95%CI -48, 56], p=0.876 |
| Goh^27^  (2019) | Malaysia | CS | 53 (40) | 35/18 | 63, median | Median 19.5, IQR 6.8-55.0 mo | NR | FSS-9 (sum)**, Median=32  (IQR=22-40),  34% | 10mgs,  Median 0.61, IQR 0.34-0.84  m/s | Spearman correlation,  rho=-0.05, [95%CI= -0.37, 0.27], p=0.77 |
| Hamre^3^  (2021) | Norway | P | 101 | 80/21 | 56 | 12 mo | I | FSS-9 (avg)**,  Median=2.8 (IQR=1.8-4.4),  NR | HUNT-3 physical activity,  Median 2.17, SD 0.37 | Multiple linear regression,  B=-0.07, [95%CI= -0.12, -0.02], p=0.004 |
| Hei Chow^28^ | Australia | CS | 23(20) | 13/10 | 75 | Mean 35, range 7-77 d | H+I | FAS,  Mean=22 (SD 7.43)  NR | GENEActive, total PA time, Mean 99, SD 74 min/d | Multiple linear regression, r^2^=0.052, [95%CI= -7,08, 2.590], p=0.333 |
| Ho^4^  (2021) | Hong Kong | CS | 112 | 74/38 | 64 | Mean 6.08, SD 4.80 y | NR | FAS,  Mean=22.72 (SD=6.19), 52.7% | 5STS,  Mean 17.58, SD 7.8, seconds | Pearson correlation,  r=0.11, p=0.27 |
| Hoang^5^  (2012) | France | CS | 32 | 21/11 | 65 | Mean 40, SD 42.2 mo | H+I | FSS-9 (avg)**,  Mean=4.3 (SD=1.8),  66% | 10mgs,  Mean 0.60, SD 0.3  m/s | Student’s t-test (or non-parametric equivalent if appropriate), p>0.05 |
|  | France | CS | 32 | 21/11 | 65 | Mean 40, SD 42.2 mo | H+I | FSS-9 (avg)**,  Mean=4.3 (SD=1.8),  66% | Dijon PA Score | Student’s t-test (or non-parametric equivalent if appropriate), p>0.05 |
| Kirchberger^6^ | Germany | P | 505(422) at 3 mo | 293/212  at 3 mo | 69  at 3 mo | 0,3,12 mo | I+H | FAS,  NR, no fatigue=69%, moderate fatigue=25%, severe=6%, at 3 mo | IPAQ-total MET min/w, NA | Multiple linear regression,  Β=-0,0004, [95%CI=-0,0006, 0,00009], p=0,0089 |
| Lerdal^7^  (2011) | Norway | CS | 115 | 68/47 | 68 | Mean 4.6, SD 3.2 d | H+I | FSS-9,  NR,  57% | SF-36pf,  Mean 58.90, SD 33.3 | Pearson correlation,  r=-0.40, p<0.05 |
| Lewis^8^  (2011) | UK | RCT | 66(58) | 36/30 | 71 | Median 60, IQR 84-280 d | H+I | SF-36v,  Mean=53.60 (SD=20.6),  NR | Lower Limb Extensor Power (unaffected leg),  Median 1.05, IQR 0.47-1.55 W/kg | Pearson correlation,  r=0.38, p=0.003 |
| Mahendran^9^  (2020) | Australia | P | 36 | 25/11 | 71 | 6 mo | NR | FSS-7 (avg)**,  Median=4 (IQR=2-4),  NR | ActivPAL,  Mean 4946, SD 3732, steps/d *at 6 months* | Spearman correlation,  rho=-0.06, p>0.05 |
| Michael^10^*  (2007) | US | CS | 79 | 42/37 | 65 | Median 10.3, range 6-166 mo | I | FSS-9 (avg)**, Mean= 3.28, (SD=1.36)  42% | Step activity monitor,  Mean 1389, SD 798, range 256-4476, Steps/d | Pearson correlation,  r=0.170, p>0.05 |
| Michael  (2006) | US | CS | 53 | 31/22 | 66 | Median 10.3, range 6-166 mo | I | FSS-9 (avg)**,  Mean=3.9  42% | 10mgs,  NR | Multiple linear regression, B=-0.087, p=0.622 |
| Miller^11^  (2013) | US | CS | 77 | 58/19 | 64 | >6 mo | H+I | FSS-9 (avg)**,  NR,  66% (>4points) | 10mgs(fast),  Mean 1.32, SD 0.64, m/s | Pearson correlation,  r=-0.175, p=0.128 |
| Muci^29^  (2020) | Turkey | CS | 37 | 25/12 | 54 | Median 4, range 1-45 mo | H+I | FSS-9 (sum)**, Mean=30.27 (SD=16.73),  NR | 10mgs,  Mean 0.73, SD 0.33, m/s | Pearson correlation,  r=-0.343, p=0.038 |
| Obembe^30^  (2014) | Nigeria | CS | 70 | 41/29 | 54 | mean 12.4, SD 7.6 mo | H+I | mFIS,  Mean=37.9 (SD=11.7), range 6-61,  NR | 10mgs,  Mean 0.32, SD 0.23, m/s | Spearman correlation, rho=0.227, p=0.059 |
| Oyake^12^  (2021) | Japan | CS | 23 | 17/6 | 60 | Mean 69.7, SD 30.2 d | H+I | FSS-9 (sum)**, Median=32 (IQR=27-42), NR | VO_2_peak,  Mean 18.0, SD 4.2, mL/kg/min | Spearman correlation,  rho=-0.264, p=0.224 |
| Paul^31^  (2016) | UK | CS | 22 | 10/12 | 56 | Mean 4.2, SD 4.0 y | NR | FSS-9 (avg)**,  Mean 3.8, SD 1.3,  NR | ActivPAL,  Mean 1.0, SD 0.6, hours stepping with cadence >20 | Spearman correlation, rho=0.12, p=0.595 |
| Pedersen^13^ | Sweden | P | 430(425) | 282/148 | 57 | 7 years | I | D-FIS,  Mean 8.3, SD7.4,  NR | SGPALS,  NR,  Low PA (less than 4 hours moderate PA/w=15% | Spearman correlation,  roh=0.20, p<0.001 |
| Robinson^14^  (2011) | US | CS | 50(46) | 27/23 | 46 | Mean 85, SD 89.9 mo | NR | FSS-9 (sum)**,  Mean=36.1 (SD=15.8), 48% | VKR TwinStep Pedometer,  Mean 2540, SD 2176, steps per day | Pearson correlation,  r=-0.38, p=0.01 |
| Sánchez-Sánchez^15^ (2021) | Spain | CS | 61(57) | 37/24 | 58 | Median 64, IQR 38.5-105.5 mo | H+I | FSS-9 (avg)**,  Mean=3.5 (SD 1.7), 35.1% | ActiGraph,  Mean 0.87, SD 0.41, % wear time in MVPA | Multiple linear regression, β=-0.140, p=0.217 |
| Schüler^16^  (2021) | Germany | CS | 67(65) | 36 | 55 | Mean 5.5, SD 5.08 y | NR | FSMC mean 3.21, SD 0.93,  NR | Physical activity, exercise, and sport Questionnaire  Mean 358.44, SD 632.26, physical activity min/d | Kendall’s Tau,  τ= -0.116, p=0.182 |
| Shaughnessy^32^  (2006) | US | CS | 312(305) | 127 | 63 | Mean 60.2, SD 48.1 mo | NR | Fatigue influence on daily activities, 68%, 3% missing | Exercise behavior,  >1 time/week, 57%, 1% missing | Pearson correlation,  r=-0.100, p=0.081 |
| Shepherd^33^  (2018) | UK | RCT | 41 | 27 | 70 | Mean 60, SD 47 mo | H+I | FAS,  Mean=26 (SD=7),  NR | GENEActive accelerometer,  Mean 54, SD 56, min in MVPA | Multiple linear regression, B=0.02, [95%CI= -0.03,  -0.07], p=0.46 |
| Sibbritt^34^ | Australia | CS | 576(534) | 316/260 | 76 | Mean 10.4, SD 8.9 y | NR | MFIS-5,  NR,  NR | METs/week, NR, cases %:  Inactive=38%, moderately active=17%, highly active=45% | Multiple logistic regression, OR=0.94, [95%CI=0.89, 0.99], p=0.025 |
| Tang^35^  (2010) | Hong Kong | CS | 458 | 282 | 66 | 3 mo | I | FSS-9 (avg)**,  Mean=3.1 (SD=1.4), NR | SF-36pf,  Mean 71.5, SD 27.9 | Pearson correlation,  r=-0.202, p>0.05 |
| Tseng^36^  (2010) | US | CS | 21 | 12 | 60 | Mean 4.1, SD 3.5 y | H+I | FSS-9 (avg)**, Mean=4.2 (SD=1.7), NR | VO_2_peak,  Mean 16.2, SD 6.5 mL/kg/min | Pearson correlation,  r=-0.125, p>0.05 |
| van de Port^37^  (2008) | Netherlands | CS | 102(72) | 46 | 59 | 3 y | H+I | FSS-9,  NR,  46% | Community ambulation questionnaire,  74% unlimited community walkers | Multiple logistic regression, OR=-0.994, p>0.05 |
| Wang^38^  (2014) | China | CS | 265 | 151 | 63 | 18 mo | I | FSS-9 (avg)**, Mean=3.5 (SD 1.6), 40% | Pre-stroke exercise,  71.3% exercised | Multiple logistic regression, OR=4.01, [95%CI= 1.95, 8.24], p<0.001 |

* main study

** FSS is reported either as a sum score (sum) or as an average score (avg) calculated from the sum score

β=standardized beta, B=unstandardized beta, CS=Cross-sectional, d=days, FAS=Fatigue Assessment Scale, FIS=Fatigue Impact Scale, FSMC=Fatigue Scale for Motor and Cognitive Functions, FSS=Fatigue Severity Scale, H=hemorrhagic, I=ischemic, IPAQ=International Physical Activity Questionnaire, IQR=interquartile range, kg=kilograms, MFIS-5=5-item Modified Fatigue Impact Scale, min=minutes, mL=milliliters, mo=months, m/s=meter/second, MVPA=moderate-to-vigorous physical activity, NR=not reported, OR=odds ratio, P=Prospective, PA=physical activity, RCT=Randomized controlled trial, SD=standard deviationp=p-value, SF-36pf=Short Form-36 physical function, SF-36v=Short Form-36 vitality, SGPALS= Saltin–Grimby Physical Activity Level Scale, VO_2_peak=peak oxygen uptake, w=weeks, y=years, 10mgs=10-meter gait speed, 95%CI=95% confidence interval

**Table S6. Direction of association for fatigue and physical activity/fitness, n=24**

| **Studies** | **Direction^*^** |
| --- | --- |
| Physical activity n=17 | |
| Almhdawi^23^ 2021 | negative |
| Danks^24^ 2016 | neutral |
| Duncan^1^ 2015 | negative |
| Egerton^26^ 2015 | positive |
| Fini^2^ 2021 | positive |
| Hamre^3^ 2021 | negative |
| Hei Chow^28^ 2021 | negative |
| Hoang^5^ 2012 | negative |
| Kirchberger^6^ 2022 | negative |
| Mahenderan^9^ 2020 | negative |
| Paul^31^ 2016 | positive |
| Pedersen^13^ 2022 | negative |
| Sánchez-Sánchez^15^ 2021 | negative |
| Shepherd^33^ 2018 | positive |
| Schüler^16^ 2020 | negative |
| Sibbritt^34^ 2022 | negative |
| Wang^38^ 2014 | negative |
| Physical fitness n=7 | |
| Durcan^25^ 2016 | negative |
| Goh^27^ 2019 | negative |
| Hoang^5^ 2012 | neutral |
| Michael^10^ 2007 | negative |
| Obembe^30^ 2014 | positive |
| Oyake^12^ 2021 | negative |
| van de Port^37^ 2008 | negative |

| positive | neutral | negative |
| --- | --- | --- |

* Direction: negative=higher fatigue associated with reduced physical activity/fitness levels; positive=higher fatigue associated with higher physical activity/fitness levels; neutral=no association/direction of effect

**Table S7. Risk of Bias assessment results**

| Study/year | | Study participation | Study Attrition | Prognostic factor (Fatigue) | Outcome  (Physical activity/fitness) | Study confounding | Statistical analysis | Overall Risk of Bias |
| --- | --- | --- | --- | --- | --- | --- | --- | --- |
| Almhdawi^23^ (2021) | | Moderate | NA | Low | High | Low | Low | High |
| Danks^24^ (2017) | | High | NA | Low | Low | Moderate | Low | High |
| Duncan^1^ (2015) | | Moderate | Moderate | Low | Moderate | Low | Low | Moderate |
| *Durcan^25^ (2016)* | | High | NA | Low | Low | High | Low | High |
| Egerton^26^ (2015) | | Low | High | High | High | Low | Low | High |
| Fini^2^ (2021) | | Moderate | Low | Low | Low | Low | Low | Moderate |
| *Goh^27^ (2019)* | | Moderate | NA | Low | Moderate | High | Low | High |
| Hamre^3^ (2021) | | Low | High | Low | Low | Moderate | Low | High |
| Hei Chow^28^ (2021) | | Moderate | NA | Low | Low | Low | Low | Moderate |
| *Ho^4^ (2021)** | | Moderate | NA | Low | Low | Low | Low | Moderate |
| Hoang^5^ (2012) | *Hoang^5^*  *(2012)* | Low | NA | Low | Low | Low | Low | Low |
| Kirchberger^6^ (2022) | | Moderate | High | Moderate | Moderate | Low | Low | High |
| *Lerdal^7^ (2011)** | | Low | NA | Low | Low | Low | Low | Low |
| *Lewis^8^ (2011)** | | Moderate | NA | Low | Low | Low | Low | Moderate |
| Mahendran^9^ (2020) | | Low | Low | Low | Low | High | Low | High |
| Michael^10^ (2007)* | *Michael^10^ (2007)* | Moderate | NA | Low | Low | High | Low | High |
| *Miller^11^ (2013)** | | High | NA | Low | Low | High | Low | High |
| *Muci^29^ (2020)** | | Low | NA | Low | Low | High | Low | High |
| *Obembe^30^ (2014)* | | Moderate | NA | Low | Low | High | Low | High |
| *Oyake^12^ (2021)* | | Moderate | NA | Low | Low | Low | Low | Moderate |
| Paul^31^ (2016) | | High | NA | Low | Low | High | Low | High |
| Pedersen^13^ (2022) | | Low | Moderate | Low | Low | Low | Low | Moderate |
| Robinson^14^ (2011)* | | Low | Low | Low | Low | High | Low | High |
| Sánchez-Sánchez^15^ (2021) | | Moderate | NA | Low | Low | Low | Low | Moderate |
| Schüler^16^ (2021) | | High | NA | Low | Low | High | Low | High |
| Shaughnessy^32^ (2006)* | | High | NA | Moderate | Moderate | High | Low | High |
| Shepherd^33^ (2018) | | Moderate | NA | Low | Low | Low | Low | Moderate |
| Sibbritt^34^ (2022) | | Moderate | NA | Low | Low | Moderate | Low | Moderate |
| *Tang^35^ (2010)** | | Low | NA | Low | Low | Low | Low | Low |
| *Tseng^36^ (2010)** | | Moderate | NA | Low | Low | Moderate | Low | Moderate |
| *van de Port^37^ (2008)* | | Moderate | NA | Low | Moderate | Low | Low | Moderate |
| Wang^38^ (2014) | | Low | NA | Low | Moderate | Moderate | Low | Moderate |
|  | |  |  |  |  |  |  |  |
| Overall risk of bias for **all studies reporting** **physical activity** (n=20): Low n=1, Moderate n=8, High n=11 | | | | | | | | |
| Overall risk of bias for **physical activity** **meta-analysis** studies (n=3): Low n=0, Moderate n=0 , High n=3 | | | | | | | | |
| Overall risk of bias for **physical activity** **vote-counting** studies (n=16): Low n=1, Moderate n=8, High n=7 | | | | | | | | |
| Overall risk of bias for **all studies reporting** **physical fitness** (n=14): Low n=3, Moderate n=5, High n=6 | | | | | | | | |
| Overall risk of bias for **physical fitness** **meta-analysis studies** (n=7): Low n=2, Moderate n=3 , High n=2 | | | | | | | | |
| Overall risk of bias for **physical fitness vote-counting** studies (n=7): Low n=1, Moderate n=2 , High n=4 | | | | | | | | |

| Low risk of bias | Moderate risk of bias | High Risk of Bias |
| --- | --- | --- |

* denotes studies included in a meta-analysis

First column: white background/no colour=physical activity studies; dark grey/Italics=physical fitness studies; note Hoang and Michael contributed both outcomes.

**Table S8. Grading of recommendation, assessment, development and evaluation for meta-analyses and vote-counting analyses separately**

| **№ of studies** | **Certainty assessment** | | | | | | **Certainty** |
| --- | --- | --- | --- | --- | --- | --- | --- |
|  | **Study design** | **Risk of bias** | **Inconsistency** | **Indirectness** | **Imprecision** | **Other considerations** |  |
| **Physical activity (meta-analysis)** | | | | | | | |
| 3 | observational studies | serious^a^ | very serious^b^ | not serious | serious^c^ | none | ⨁◯◯◯ Very low |
| **Physical activity (vote counting)** | | | | | | | |
| 17 | observational studies | serious^d^ | Serious^e^ | not serious | Serious^f^ | none | ⨁◯◯◯ Very low |
| **Physical fitness (meta-analysis)** | | | | | | | |
| 7 | observational studies | Serious^g^ | not serious | not serious | not serious | none | ⨁⨁⨁◯ Moderate |
| **Physical fitness (vote counting)** | | | | | | | |
| 7 | observational studies | Serious^h^ | serious^i^ | not serious | Serious^j^ | none | ⨁◯◯◯ Very low |

#### Explanations

a. Risk of Bias: All studies had a high risk of bias in at least one domain.

b. Inconsistency: Different directions of effect in at least one study. High I^2,^ and prediction intervals could not be calculated.

c. Just over 400 participants, moderately large 95% confidence intervals, (different direction of effect).

d. Risk of Bias: Eight studies had a high risk of bias in one domain or more and only one study with low overall risk of bias.

e. Inconsistency: Twelve studies had the same direction of association, one showed no direction of association, and 4 hade the opposite direction of association.

f. Imprecision: Varying results when it comes to statistical significance.

g. Risk of Bias: Two studies with a high risk of bias in at least one domain. Only one study with low overall risk of bias.

h. Inconsistency: Four studies had a high risk of bias in at least one domain. Only one study had low overall risk of bias.

i. Inconsistency: Five studies had the same direction of association, one showed no association and one had the opposite direction of association.

j. Imprecision: Less than 400 participants across studies.

**Table S9. Grading of recommendation, assessment, development and evaluation for meta-analyses and vote counting analyses combined**

| **№ of studies** | **Certainty assessment** | | | | | | **Certainty** |
| --- | --- | --- | --- | --- | --- | --- | --- |
|  | **Study design** | **Risk of bias** | **Inconsistency** | **Indirectness** | **Imprecision** | **Other considerations** |  |
| **Physical activity** | | | | | | | |
| 20 | observational studies | serious^a^ | very serious^b^ | not serious | not serious^c^ | none | ⨁◯◯◯ Very low |
| **Physical fitness** | | | | | | | |
| 14 | observational studies | serious^d^ | serious^e^ | not serious | not serious^f^ | none | ⨁⨁◯◯ Low |

#### Explanations

a. Risk of bias: Ten studies had high risk of bias in at least one domain. Only one study had low overall risk of bias.

b. Inconsistency: High I^2,^ and prediction intervals could not be calculated. The direction and magnitude of association varied across studies.

c. Imprecision: Not serious, borderline. Total number of participants across studies was about 1600. The majority of the studies were small studies, many reported statistically non-significant results.

d. Risk of bias: Six studies had high risk of bias in at least one domain. Two studies had low overall risk of bias.

e. Inconsistency: Twelve studies had the same direction of association; one showed no association and one showed the opposite direction of association compared to the first 12.

f. Imprecision: Not serious, borderline. Total number of participants across studies was about 1200. The majority of the studies were small studies reporting statistically non-significant results.

**References**

1. Duncan F, Lewis SJ, Greig CA, et al. Exploratory longitudinal cohort study of associations of fatigue after stroke. *Stroke* 2015; 46: 1052-1058. DOI: h<ttps://dx.doi.org/10.1161/STROKEAHA.114.008079.>

2. Fini NA, Bernhardt J, Churilov L, et al. A 2-Year Longitudinal Study of Physical Activity and Cardiovascular Risk in Survivors of Stroke. *Phys Ther* 2021; 101: 1-9.

3. Hamre C, Fure B, Helbostad JL, et al. Factors Associated with Level of Physical Activity After Minor Stroke. *J Stroke Cerebrovasc Dis* 2021; 30: 105628. DOI: 10.1016/j.jstrokecerebrovasdis.2021.105628.

4. Ho LYW, Lai CKY, Ng SSM. Contribution of sleep quality to fatigue following a stroke: a cross-sectional study. *BMC Neurol* 2021; 21: 1-9. DOI: 10.1186/s12883-021-02174-z.

5. Hoang CL, Salle JY, Mandigout S, et al. Physical factors associated with fatigue after stroke: an exploratory study. *Top Stroke Rehabil* 2012; 19: 369-376. DOI: h<ttps://dx.doi.org/10.1310/tsr1905-369.>

6. Kirchberger I, Wallner F, Linseisen J, et al. Factors Associated With Early and Late Post-stroke Fatigue in Patients With Mild Impairment. Results From the Stroke Cohort Study Augsburg. *Frontiers in neurology [electronic resource]* 2022; 13: 852486. DOI: doi:h<ttps://dx.doi.org/10.3389/fneur.2022.852486.>

7. Lerdal A, Bakken LN, Rasmussen EF, et al. Physical impairment, depressive symptoms and pre-stroke fatigue are related to fatigue in the acute phase after stroke. *Disabil Rehabil* 2011; 33: 334-342. DOI: h<ttps://dx.doi.org/10.3109/09638288.2010.490867.>

8. Lewis SJ, Barugh AJ, Greig CA, et al. Is fatigue after stroke associated with physical deconditioning? A cross-sectional study in ambulatory stroke survivors. *Arch Phys Med Rehabil* 2011; 92: 295-298. DOI: h<ttps://dx.doi.org/10.1016/j.apmr.2010.10.030.>

9. Mahendran N, Kuys SS, Brauer SG. Which impairments, activity limitations and personal factors at hospital discharge predict walking activity across the first 6 months poststroke? *Disabil Rehabil* 2020; 42: 763-769. DOI: h<ttps://dx.doi.org/10.1080/09638288.2018.1508513.>

10. Michael K, Macko RF. Ambulatory activity intensity profiles, fitness, and fatigue in chronic stroke. *Top Stroke Rehabil* 2007; 14: 5-12. DOI: 10.1310/tsr1402-5.

11. Miller KK, Combs SA, Van Puymbroeck M, et al. Fatigue and pain: relationships with physical performance and patient beliefs after stroke. *Top Stroke Rehabil* 2013; 20: 347-355. DOI: h<ttps://dx.doi.org/10.1310/tsr2004-347.>

12. Oyake K, Baba Y, Suda Y, et al. Cardiorespiratory responses to exercise related to post-stroke fatigue severity. *Sci Rep* 2021; 11: 12780. DOI: h<ttp://dx.doi.org/10.1038/s41598-021-92127-w.>

13. Pedersen A, Almkvist E, Holmegaard L, et al. Fatigue 7 years post-stroke: Predictors and correlated features. *Acta Neurol Scand* 2022; 146: 295-303. DOI: doi:h<ttps://dx.doi.org/10.1111/ane.13665.>

14. Robinson CA, Shumway-Cook A, Ciol MA, et al. Participation in community walking following stroke: subjective versus objective measures and the impact of personal factors. *Phys Ther* 2011; 91: 1865-1876. DOI: h<ttps://dx.doi.org/10.2522/ptj.20100216.>

15. Sanchez-Sanchez ML, Arnal-Gomez A, Cortes-Amador S, et al. Association of Barriers, Fear of Falling and Fatigue with Objectively Measured Physical Activity and Sedentary Behavior in Chronic Stroke. *J Clin Med* 2021; 10. DOI: h<ttps://dx.doi.org/10.3390/jcm10061320.>

16. Schuler J, Wolff W, Pfeifer J, et al. The Role of Perceived Energy and Self-Beliefs for Physical Activity and Sports Activity of Patients With Multiple Sclerosis and Chronic Stroke. *Front Psychol* 2020; 11: 570221. DOI: h<ttps://dx.doi.org/10.3389/fpsyg.2020.570221.>

17. Hayden JA, van der Windt DA, Cartwright JL, et al. Assessing bias in studies of prognostic factors. *Ann Intern Med* 2013; 158: 280-286.

18. Fini NA, Bernhardt J, Holland AE. What is the relationship between physical activity and cardiovascular risk factors in stroke survivors post completion of rehabilitation? Protocol for a longitudinal study. *BMJ Open* 2017; 7: e019193.

19. Oyake K. Physiological mechanisms associated with fatigue in individuals with stroke - Relationship between post-stroke fatigue and cardiorespiratory fitness (Study Protocol). h<ttps://trialsearch.who.int/Trial2.aspx?TrialID=JPRN-UMIN000029953,> 2017.

20. Lejeune T. Functional Characterisation of Post-stroke Fatigue (Study Protocol). h<ttps://clinicaltrials.gov/ct2/history/NCT04277234,> 2020.

21. Hubacher M, Calabrese P, Bassetti C, et al. Assessment of post-stroke fatigue: the fatigue scale for motor and cognitive functions. *Eur Neurol* 2012; 67: 377-384.

22. Lerdal A, Lee KA, Bakken LN, et al. The Course of Fatigue during the First 18 Months after First-Ever Stroke: A Longitudinal Study. *Stroke Res Treat* 2012; 2012: 126275.

23. Almhdawi KA, Jaber HB, Khalil HW, et al. Post-stroke fatigue level is significantly associated with mental health component of health-related quality of life: a cross-sectional study. *Qual Life Res* 2021; 30: 1165-1172. DOI: h<ttp://dx.doi.org/10.1007/s11136-020-02714-z.>

24. Danks KA, Pohlig RT, Roos M, et al. Relationship Between Walking Capacity, Biopsychosocial Factors, Self-efficacy, and Walking Activity in Persons Poststroke. *J Neurol Phys Ther* 2016; 40: 232-238. DOI: h<ttps://dx.doi.org/10.1097/NPT.0000000000000143.>

25. Durcan S, Flavin E, Horgan F. Factors associated with community ambulation in chronic stroke. *Disabil Rehabil* 2016; 38: 245-249. DOI: h<ttps://dx.doi.org/10.3109/09638288.2015.1035460.>

26. Egerton T, Hokstad A, Askim T, et al. Prevalence of fatigue in patients 3 months after stroke and association with early motor activity: a prospective study comparing stroke patients with a matched general population cohort. *BMC Neurol* 2015; 15: 181. DOI: h<ttps://dx.doi.org/10.1186/s12883-015-0438-6.>

27. Goh HT, Stewart JC. Poststroke Fatigue Is Related to Motor and Cognitive Performance: A Secondary Analysis. *J Neurol Phys Ther* 2019; 43: 233-239. DOI: h<ttps://dx.doi.org/10.1097/NPT.0000000000000290.>

28. Hei Chow C, Fraysse F, Hillier S. The relationship between sleep and physical activity in an in-patient rehabilitation stroke setting: a cross-sectional study. *Top Stroke Rehabil* 2021: 1-10. DOI: doi:h<ttps://dx.doi.org/10.1080/10749357.2021.2006982.>

29. Muci B, Keser I, Meric A, et al. What are the factors affecting dual-task gait performance in people after stroke? *Physiother* 2020: 1-8. DOI: h<ttps://dx.doi.org/10.1080/09593985.2020.1777603.>

30. Obembe AO, Olalemi AE, Loto BO. Fatigue impact, gait and balance performance in chronic stroke survivors. *Physiother Pract Res* 2014; 35: 49-54. DOI: 10.3233/PPR-130029.

31. Paul L, Brewster S, Wyke S, et al. Physical activity profiles and sedentary behaviour in people following stroke: A cross-sectional study. *Disability and Rehabilitation: An International, Multidisciplinary Journal* 2016; 38: 362-367. DOI: 10.3109/09638288.2015.1041615.

32. Shaughnessy M, Resnick BM, Macko RF. Testing a model of post‐stroke exercise behavior. *Rehabil Nurs* 2006; 31: 15-21.

33. Shepherd AI, Pulsford R, Poltawski L, et al. Physical activity, sleep, and fatigue in community dwelling Stroke Survivors. *Sci Rep* 2018; 8: 7900. DOI: h<ttps://dx.doi.org/10.1038/s41598-018-26279-7.>

34. Sibbritt D, Peng W, Hosseini M, et al. An examination of modifiable risk factors in stroke survivors, with a view to recurrent stroke prevention. *J Stroke Cerebrovasc Dis* 2022; 31: 106547.

35. Tang WK, Lu JY, Chen YK, et al. Is fatigue associated with short-term health-related quality of life in stroke? *Arch Phys Med Rehabil* 2010; 91: 1511-1515. DOI: h<ttps://dx.doi.org/10.1016/j.apmr.2010.06.026.>

36. Tseng BY, Billinger SA, Gajewski BJ, et al. Exertion fatigue and chronic fatigue are two distinct constructs in people post-stroke. *Stroke* 2010; 41: 2908-2912. DOI: h<ttps://dx.doi.org/10.1161/STROKEAHA.110.596064.>

37. van de Port IG, Kwakkel G, Lindeman E. Community ambulation in patients with chronic stroke: how is it related to gait speed? *J Rehabil Med* 2008; 40: 23-27. DOI: h<ttps://dx.doi.org/10.2340/16501977-0114.>

38. Wang SS, Wang JJ, Wang PX, et al. Determinants of fatigue after first-ever ischemic stroke during acute phase. *PLoS ONE [Electronic Resource]* 2014; 9: e110037. DOI: <https://dx.doi.org/10.1371/journal.pone.0110037>.

| **Section and Topic** | **Item #** | **Checklist item** | **Reported (Yes/No)** |
| --- | --- | --- | --- |
| **TITLE** | | |  |
| Title | 1 | Identify the report as a systematic review. | yes |
| **BACKGROUND** | | |  |
| Objectives | 2 | Provide an explicit statement of the main objective(s) or question(s) the review addresses. | yes |
| **METHODS** | | |  |
| Eligibility criteria | 3 | Specify the inclusion and exclusion criteria for the review. | yes |
| Information sources | 4 | Specify the information sources (e.g. databases, registers) used to identify studies and the date when each was last searched. | yes |
| Risk of bias | 5 | Specify the methods used to assess risk of bias in the included studies. | yes |
| Synthesis of results | 6 | Specify the methods used to present and synthesise results. | yes |
| **RESULTS** | | |  |
| Included studies | 7 | Give the total number of included studies and participants and summarise relevant characteristics of studies. | yes |
| Synthesis of results | 8 | Present results for main outcomes, preferably indicating the number of included studies and participants for each. If meta-analysis was done, report the summary estimate and confidence/credible interval. If comparing groups, indicate the direction of the effect (i.e. which group is favoured). | yes |
| **DISCUSSION** | | |  |
| Limitations of evidence | 9 | Provide a brief summary of the limitations of the evidence included in the review (e.g. study risk of bias, inconsistency and imprecision). | yes |
| Interpretation | 10 | Provide a general interpretation of the results and important implications. | yes |
| **OTHER** | | |  |
| Funding | 11 | Specify the primary source of funding for the review. | yes |
| Registration | 12 | Provide the register name and registration number. | yes |

*From:*  Page MJ, McKenzie JE, Bossuyt PM, Boutron I, Hoffmann TC, Mulrow CD, et al. The PRISMA 2020 statement: an updated guideline for reporting systematic reviews. BMJ 2021;372:n71. doi: 10.1136/bmj.n71

For more information, visit: <http://www.prisma-statement.org/>

| **Section and Topic** | **Item #** | **Checklist item** | **Location where item is reported** |
| --- | --- | --- | --- |
| **TITLE** | | | Page |
| Title | 1 | Identify the report as a systematic review. | 1 |
| **ABSTRACT** | | |  |
| Abstract | 2 | See the PRISMA 2020 for Abstracts checklist. | 2 |
| **INTRODUCTION** | | |  |
| Rationale | 3 | Describe the rationale for the review in the context of existing knowledge. | 4 |
| Objectives | 4 | Provide an explicit statement of the objective(s) or question(s) the review addresses. | 5 |
| **METHODS** | | |  |
| Eligibility criteria | 5 | Specify the inclusion and exclusion criteria for the review and how studies were grouped for the syntheses. | 5 |
| Information sources | 6 | Specify all databases, registers, websites, organisations, reference lists and other sources searched or consulted to identify studies. Specify the date when each source was last searched or consulted. | 5 |
| Search strategy | 7 | Present the full search strategies for all databases, registers and websites, including any filters and limits used. | Fig S1 |
| Selection process | 8 | Specify the methods used to decide whether a study met the inclusion criteria of the review, including how many reviewers screened each record and each report retrieved, whether they worked independently, and if applicable, details of automation tools used in the process. | 6 |
| Data collection process | 9 | Specify the methods used to collect data from reports, including how many reviewers collected data from each report, whether they worked independently, any processes for obtaining or confirming data from study investigators, and if applicable, details of automation tools used in the process. | 6 |
| Data items | 10a | List and define all outcomes for which data were sought. Specify whether all results that were compatible with each outcome domain in each study were sought (e.g. for all measures, time points, analyses), and if not, the methods used to decide which results to collect. | 6,  Table S5 |
|  | 10b | List and define all other variables for which data were sought (e.g. participant and intervention characteristics, funding sources). Describe any assumptions made about any missing or unclear information. | 6,  Table S5 |
| Study risk of bias assessment | 11 | Specify the methods used to assess risk of bias in the included studies, including details of the tool(s) used, how many reviewers assessed each study and whether they worked independently, and if applicable, details of automation tools used in the process. | 7 |
| Effect measures | 12 | Specify for each outcome the effect measure(s) (e.g. risk ratio, mean difference) used in the synthesis or presentation of results. | Table S5 |
| Synthesis methods | 13a | Describe the processes used to decide which studies were eligible for each synthesis (e.g. tabulating the study intervention characteristics and comparing against the planned groups for each synthesis (item #5)). | 6,7 |
|  | 13b | Describe any methods required to prepare the data for presentation or synthesis, such as handling of missing summary statistics, or data conversions. | NA |
|  | 13c | Describe any methods used to tabulate or visually display results of individual studies and syntheses. | 6,7 |
|  | 13d | Describe any methods used to synthesize results and provide a rationale for the choice(s). If meta-analysis was performed, describe the model(s), method(s) to identify the presence and extent of statistical heterogeneity, and software package(s) used. | 6,7 |
|  | 13e | Describe any methods used to explore possible causes of heterogeneity among study results (e.g. subgroup analysis, meta-regression). | 6 (NA) |
|  | 13f | Describe any sensitivity analyses conducted to assess robustness of the synthesized results. | 6 (NA) |
| Reporting bias assessment | 14 | Describe any methods used to assess risk of bias due to missing results in a synthesis (arising from reporting biases). | 7 |
| Certainty assessment | 15 | Describe any methods used to assess certainty (or confidence) in the body of evidence for an outcome. | 7 |
| **RESULTS** | | |  |
| Study selection | 16a | Describe the results of the search and selection process, from the number of records identified in the search to the number of studies included in the review, ideally using a flow diagram. | 7,8, Fig S2 |
|  | 16b | Cite studies that might appear to meet the inclusion criteria, but which were excluded, and explain why they were excluded. | Fig S2 |
| Study characteristics | 17 | Cite each included study and present its characteristics. | Table S5 |
| Risk of bias in studies | 18 | Present assessments of risk of bias for each included study. | Table S7 |
| Results of individual studies | 19 | For all outcomes, present, for each study: (a) summary statistics for each group (where appropriate) and (b) an effect estimate and its precision (e.g. confidence/credible interval), ideally using structured tables or plots. | Table S5 |
| Results of syntheses | 20a | For each synthesis, briefly summarise the characteristics and risk of bias among contributing studies. | 11,  Table S7 |
|  | 20b | Present results of all statistical syntheses conducted. If meta-analysis was done, present for each the summary estimate and its precision (e.g. confidence/credible interval) and measures of statistical heterogeneity. If comparing groups, describe the direction of the effect. | 9,10, Figure 1, Figure 2 |
|  | 20c | Present results of all investigations of possible causes of heterogeneity among study results. | 9-11 |
|  | 20d | Present results of all sensitivity analyses conducted to assess the robustness of the synthesized results. | NA |
| Reporting biases | 21 | Present assessments of risk of bias due to missing results (arising from reporting biases) for each synthesis assessed. | 14 |
| Certainty of evidence | 22 | Present assessments of certainty (or confidence) in the body of evidence for each outcome assessed. | 11,  Table S8, Table S9 |
| **DISCUSSION** | | |  |
| Discussion | 23a | Provide a general interpretation of the results in the context of other evidence. | 12,13 |
|  | 23b | Discuss any limitations of the evidence included in the review. | 14 |
|  | 23c | Discuss any limitations of the review processes used. | 14,15 |
|  | 23d | Discuss implications of the results for practice, policy, and future research. | 15 |
| **OTHER INFORMATION** | | |  |
| Registration and protocol | 24a | Provide registration information for the review, including register name and registration number, or state that the review was not registered. | 3 |
|  | 24b | Indicate where the review protocol can be accessed, or state that a protocol was not prepared. | 3 |
|  | 24c | Describe and explain any amendments to information provided at registration or in the protocol. | 6,8,14 |
| Support | 25 | Describe sources of financial or non-financial support for the review, and the role of the funders or sponsors in the review. | 15 |
| Competing interests | 26 | Declare any competing interests of review authors. | 16 |
| Availability of data, code and other materials | 27 | Report which of the following are publicly available and where they can be found: template data collection forms; data extracted from included studies; data used for all analyses; analytic code; any other materials used in the review. | Data extraction template (Table S1) |

*From:*  Page MJ, McKenzie JE, Bossuyt PM, Boutron I, Hoffmann TC, Mulrow CD, et al. The PRISMA 2020 statement: an updated guideline for reporting systematic reviews. BMJ 2021;372:n71. doi: 10.1136/bmj.n71

For more information, visit: <http://www.prisma-statement.org/>
